# Advanced modafinil-loaded transethosomes for brain targeting: development, ex-vivo permeation and radio-distribution evaluations

**DOI:** 10.1007/s13346-026-02103-y

**Published:** 2026-04-17

**Authors:** Marwa Eid Sayyed, Sadek Ahmed, Khaled M. Attallah

**Affiliations:** 1 Labeled Compounds Department, Hot Laboratories and Waste Management Center, Egyptian Atomic Energy Authority, P.O. Box 13759, Cairo, Egypt; 2 Department of Pharmaceutics and Industrial Pharmacy, Faculty of Pharmacy, Cairo University, Cairo, Egypt

**Keywords:** Modafinil, Transethosomes, Physicochemical, Technetium-99m, Brain/blood ratio

## Abstract

**Abstract:**

Modafinil is a well-established wake-promoting agent with emerging applications as a cognitive enhancer; however, its clinical potential is constrained by poor aqueous solubility and suboptimal systemic absorption, limiting effective brain delivery. This study presents transethosomes as a hitherto unexplored nanocarrier for modafinil and combines design-driven formulation with nuclear imaging-based biodistribution. Transethosomal vesicles were prepared using the ethanol injection method and systematically optimized through a 2^3^ factorial design employing Design-Expert^®^ software. Key formulation variables were investigated for their impact on EE%, PS, PDI, and ZP. Additionally, an in-vivo biodistribution and pharmacokinetic studies were conducted after labeling MOD with Technetium-99 m using sodium dithionite as a reducing agent. The optimized formulation achieved a high desirability value (0.919), superior EE% (85.87%), nanoscale PS (180.30 nm), and a negative ZP (− 42.60 mV), indicative of excellent vesicular stability. Morphological and FTIR analyses confirmed spherical vesicles, drug–excipient compatibility, and preservation of Modafinil’s structure. In-vitro studies demonstrated a controlled biphasic release, supporting sustained drug availability, while stability assessments revealed no significant changes in vesicular characteristics over time. Ex-vivo studies highlighted markedly enhanced permeability, due to improved membrane fluidity and vesicle deformability from ethanol and the edge activator. The radiolabeling efficiency was high (92.18%), and it was stable for two hours. Biodistribution and pharmacokinetic studies confirmed significantly higher brain drug accumulation, elevated brain C_max_ (5.4%ID/g) and AUC, reduced T_max_ (10 min) and high relative bioavailability (424.3 ± 4.5%). Importantly, histopathological examination of nasal mucosa revealed normal architecture. Collectively, these findings establish transethosomes as a promising and safe nano-platform for advanced brain targeting.

**Highlights:**

- Modafinil-loaded transethosomes were designed as nanovesicles for brain targeting.
- The optimized formulation showed spherical morphology, uniform size distribution, and strong drug–carrier compatibility.
- Stability studies confirmed preserved physicochemical properties throughout storage.
- Ex-vivo permeation revealed significantly enhanced mucosal drug transport versus plain modafinil dispersion.
- In-vivo radio-distribution unambiguously confirmed superior uptake into the brain and improved targeting efficiency when contrasted with the reference preparation.

**Graphical Abstract:**

**Figure:**
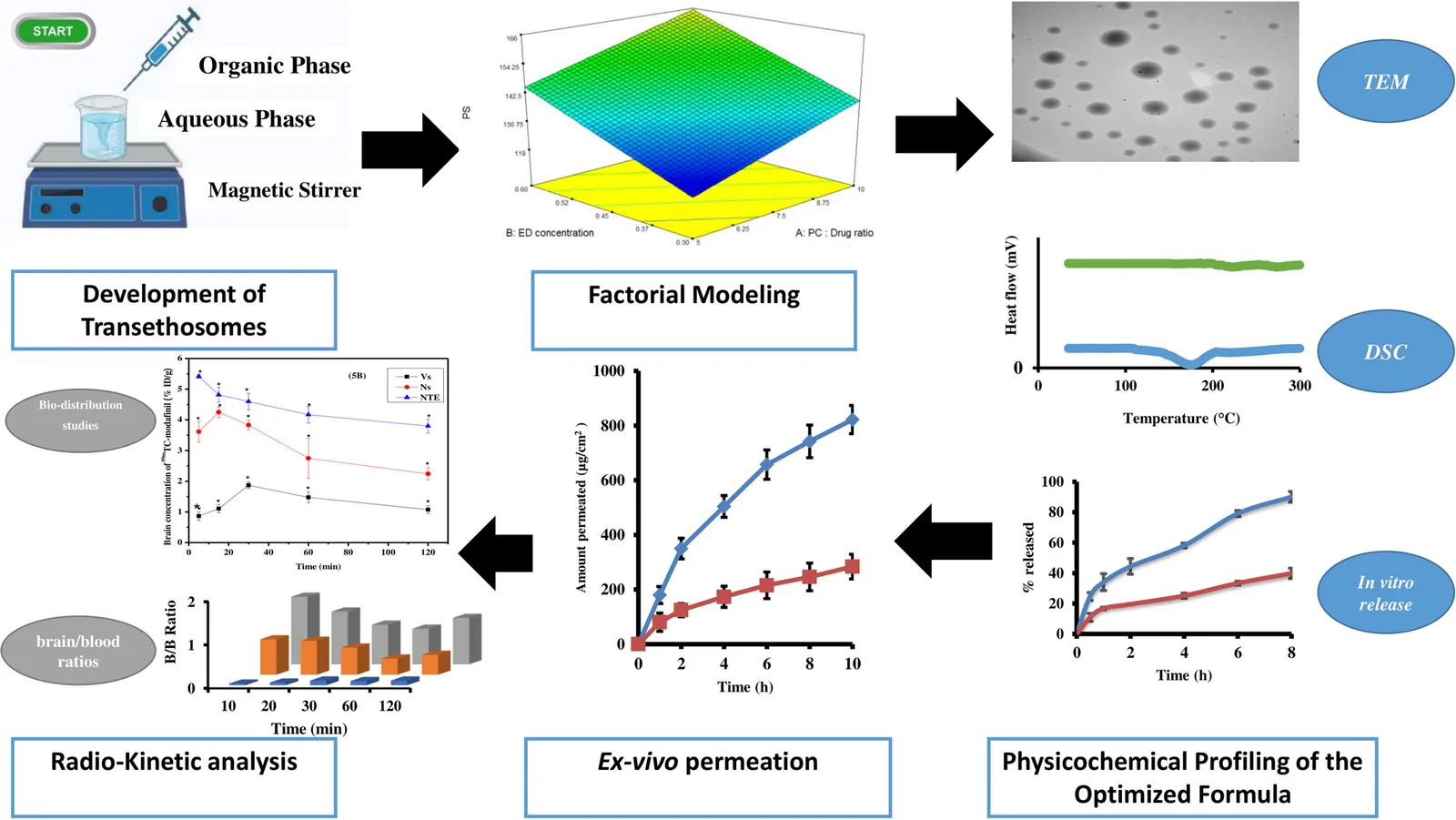

**Supplementary Information:**

The online version contains supplementary material available at https://doi.org/10.1007/s13346-026-02103-y.

## Introduction

Modafinil (MOD) is a clinically approved wake-promoting agent, originally indicated for the management of sleep-related disorders, yet it is widely recognized for its off-market utility as a cognitive enhancer [1]. Beyond improving wakefulness, studies have demonstrated its ability to enhance multiple cognitive functions, including attention, memory, executive control, and overall alertness [2]. At the neurochemical level, its activity is primarily linked to inhibition of the dopamine transporter (DAT), resulting in elevated extracellular dopamine levels followed by the agonism of the D1 and D2 dopamine receptor subtypes [3]. Furthermore, modafinil also exerts influence on the norepinephrine transporter (NET), thereby amplifying noradrenergic signaling and sustaining enhanced dopaminergic tone [4].

Despite its promising neuro-pharmacological profile, MOD is classified as a BCS Class II drug, characterized by low aqueous solubility, which limits its dissolution in gastrointestinal fluids and results in variable oral bioavailability and delayed onset of action. Furthermore, systemic administration does not ensure efficient or rapid brain targeting due to physiological barriers and first-pass metabolism. These limitations necessitate the development of an advanced delivery platform capable of enhancing solubility, improving permeation across biological membranes, and facilitating direct nose-to-brain transport. MOD has been previously formulated using various strategies, including lipid-based systems [5], complexation with β-cyclodextrin [6], and polymeric nanoparticles [7], to enhance its solubility, stability, and bioavailability. In this context, transethosomes, a highly deformable lipid vesicles enriched with ethanol and edge activators, represents a promising strategy to enhance nasal mucosal permeation, improve drug deposition, and promote efficient brain targeting of MOD.

Lipid-based delivery systems have demonstrated significant potential in overcoming solubility barriers and enhancing the uptake of hydrophobic drugs [5, 8]. Various nanosystems have been explored for direct nose-to-brain delivery, including nanosuspension [9], transferosomes [10], elastosomes [11], and polymeric micelles [12]. However, many of these systems are limited by factors such as restricted vesicle flexibility, lower drug loading, or suboptimal permeation through biological barriers. In contrast, transethosomes, as an advanced third-generation vesicular platform, combine the deformability of elastic vesicles with the presence of penetration enhancers, resulting in improved drug encapsulation, enhanced mucosal permeation, and greater vesicle stability compared with conventional ethosomes and transferosomes [13].

Transethosomes are highly deformable, vesicles composed primarily of phospholipids, edge activators (ED), ethanol, and water [14]. Their structural flexibility allows them to deform and subsequently regain their shape after crossing biological membranes, a property that greatly enhances their penetration ability [15]. Ethanol acts as both a penetration enhancer and bilayer fluidizer, while edge activators disrupt lipid packing within biological barriers, creating a synergistic effect that facilitates efficient drug transport [16, 17]. In this study, ethanol concentrations were selected within the commonly reported range for transethosomal systems (25–50% v/v), taking into consideration nasal mucosal safety. Histological analysis confirmed that the nasal mucosa of treated animals retained normal structural integrity, with no signs of epithelial disruption, inflammation, or irritation. Furthermore, the optimized formulation exhibited a pH within the physiological range of nasal secretions (4.5–6.5) [13]. The incorporation of cholesterol further stabilizes the vesicular bilayer, modulates rigidity, and contributes to controlled release, thereby reducing the need for frequent dosing and minimizing off-target toxicity [18]. Phosphatidylcholine (PC), the principal phospholipid in these carriers, plays a vital role in their performance. Attributed to its intrinsic tissue compatibility and degradation potential within the body and its abundance in eukaryotic cell membranes, phosphatidylcholine provides a safe and efficient matrix for drug encapsulation [19]. Collectively, these properties make transethosomes a promising platform for systemic and brain-targeted drug delivery [20].

Radiobiological investigations represent a cornerstone in evaluating the in-vivo performance of advanced drug delivery systems, as they allow precise monitoring of bio-distribution, pharmacokinetics, organ-specific targeting and therapeutic potential [21, 22]. In nuclear medicine, [^99m^Tc]Tc remains the radionuclide of choice on account of its exceptional combined chemical and nuclear traits which include a short physical half-life of approximately 6.02 h, along with gamma ray emission of 140 keV that is well-suited for high-resolution imaging [23–25].

Intranasal administration can provide a more rapid onset of action by bypassing gastrointestinal absorption and first-pass metabolism, reduce systemic exposure and peripheral side effects, and potentially allow for lower effective doses compared to orally administered drugs which enter the peripheral circulation directly, resulting in limited blood–brain barrier (BBB) penetration, and accordingly distributing these drugs to non-targeted areas. Moreover, nose-to-brain transport via olfactory and trigeminal pathways offers a direct route that may be particularly beneficial in acute settings or in patients with impaired oral absorption [26, 27].

In light of these factors, we postulated that, in comparison to traditional delivery systems, formulating modafinil into transethosomal vesicles would improve its solubility, permeation, and brain-targeting efficacy. The primary objective of this study was to develop and optimize MOD-loaded transethosomes using a systematic 2^3^ factorial design to investigate the influence of critical formulation factors, namely phosphatidylcholine-to-drug ratio, edge activator concentration, and ethanol content on the encapsulation efficiency, particle size, polydispersity index, and zeta potential to ensure stability and effective delivery. The optimized formula was further assessed for its ex-vivo nasal permeation and in-vivo radio-kinetic distribution studies using [^99m^Tc]Tc to evaluate its brain-targeting efficiency and pharmacokinetic behavior. Taken together, these comprehensive findings position transethosomes as a viable advanced nanocarrier for enhanced and safer brain targeting.

## Material and methods

### Materials

Modafinil with purity ≥ 98% (HPLC) was generously provided from Sigma-Aldrich (Massachusetts, USA) and used as the model drug. Fisher Scientific (Leicestershire, UK) supplied the egg yolk lecithin, which was specified to contain approximately 72% PC. BASF (Germany) supplied the edge activator, Cremophor^®^ RH 40 (polyoxyl 40 hydrogenated castor oil, USP/NF grade). Sigma-Aldrich (Massachusetts, USA) supplied the remaining materials, including cholesterol, dialysis membranes (14,000 Da, molecular weight cut-off), and sodium dithionite (Na₂S₂O₄, 174.11 g/mol). Anhydrous KH_2_PO_4_ and Na_2_HPO_4_, alongside ethanol (95% v/v), were sourced from Adwic, El-Nasr Pharmaceutical Chemicals Co. (Cairo, Egypt). The radioisotope technetium-99 m ([^99m^Tc]Tc) originated from the Egyptian Atomic Energy Authority’s Radioisotope Production Facility. Throughout all experimental protocols, freshly prepared deionized water was employed to ensure reproducibility, maintain formulation stability, and avoid ionic interference.

### Factorial modeling and experimental strategy

This investigation deployed a methodical approach to refine the MOD-loaded transethosomes system. The study utilized a 2^3^ factorial experimental layout, where a trio of formulation parameters served as the independent variables: the PC-to-drug ratio (Factor A), the concentration of the edge activator expressed as % w/v (Factor B), and the volume of ethanol incorporated during vesicle preparation (Factor C). Following the outcomes derived from preliminary screening, the independent variables were assessed at two predefined high and low levels. The key dependent responses monitored included EE% (Y₁), PS (Y₂), poly-dispersity PDI (Y₃), and ZP (Y₄). The optimization strategy aimed to yield the highest possible EE% and ZP, with the additional requirement that PS be maintained within the nanoscale range to ensure stability and effective delivery. Design-Expert^®^, a specialized statistical platform from Stat-Ease Inc. (USA), was utilized for statistical evaluation, regression model fitting, and analysis of variance (ANOVA), enabling detailed assessment of the studied factors [28, 29]. The experimental design matrix with factor levels and the applied constraints for optimization are compiled and listed in Table 1, Conversely, Table 2 comprehensively lists the specific components of every sample and their determined output metrics.

**Table 1:** Independent variables, response measures, and desirability targets in factorial design studies

Factor (independent variable)	Level	
	**−1**	** + 1**
A: PC: Drug ratio	5:1	10:1
B: ED concentration (w/v %)	0.3	0.6
C: Ethanol volume (mL)	2.5	5
**Response (dependent variable)**	**Desirability constraints**	
Y1: EE %	Maximize	
Y2: PS (nm)	In range	
Y3: PDI	Minimize	
Y4: ZP (absolute value) (mV)	Maximize	

*ED* Edge activator; *EE %* percent entrapment efficiency; *PC* phosphatidylcholine, *PDI* Poly-dispersity index, *PS* particle size, *ZP* zeta potential

**Table 2:** Assessment of responses for modafinil-entrapped transethosomes expressed as mean ± SD (*n* = 3)

Formula	Factors						
	A: PC: Drug ratio	B: ED concentration (w/v %)	C: Ethanol volume (mL)	Y1: EE % (Mean ± SD)	Y2: PS (nm) (Mean ± SD)	Y3: PDI (Mean ± SD)	Y4: ZP (mV) (Mean ± SD)
F1	5:1	0.3	5	73.56 ± 4.78	121.75 ± 1.20	0.24 ± 0.01	−22.70 ± 1.70
F2	10:1	0.6	2.5	80.65 ± 3.23	160.60 ± 0.42	0.27 ± 0.03	−31.10 ± 0.57
F3	5:1	0.6	2.5	67.15 ± 1.08	135.25 ± 1.63	0.23 ± 0.01	−23.30 ± 0.14
F4	5:1	0.3	2.5	68.24 ± 6.04	127.10 ± 1.84	0.25 ± 0.01	−19.05 ± 1.63
F5	10:1	0.6	5	85.87 ± 5.15	180.30 ± 1.70	0.27 ± 0.01	−42.60 ± 1.27
F6	10:1	0.3	2.5	77.82 ± 5.30	134.50 ± 0.14	0.16 ± 0.01	−28.75 ± 2.05
F7	10:1	0.3	5	83.76 ± 4.39	134.80 ± 2.55	0.21 ± 0.01	−30.70 ± 0.28
F8	5:1	0.6	5	74.76 ± 1.90	145.20 ± 4.10	0.27 ± 0.01	−28.65 ± 1.06

Formulation components: PC (50 or 100 mg), ED (30 or 60 mg), ethanol (2.5 or 5 mL), cholesterol (20 mg), modafinil (10 mg)

*ED*, Edge activator; *EE%*, percent entrapment efficiency; *PC*, phosphatidylcholine; *PDI*, poly-dispersity index; *PS*, particle size; *ZP*, zeta potential

### Development of MOD-entrapped transethosomes

MOD-loaded transethosomes were fabricated employing the ethanol injection technique with slight modifications [30]. Preparation of the lipid-soluble component involved dissolving 10 mg of MOD with PC) at predetermined PC: drug ratios (5:1 or 10:1), along with 20 mg of cholesterol and the selected concentration of edge activator per the formation volume (0.3% or 0.6% w/v). The cholesterol level in the transethosomes was fixed based on preliminary studies to ensure optimal vesicle stability. Cholesterol helps stabilize the bilayer, adjust membrane rigidity, and maintain structural integrity for efficient drug delivery. We introduced the components into absolute ethyl alcohol at one of two distinct volumes (2.5 or 5 mL) and held at a constant temperature of 35 °C within the water bath supplied by Crest Ultrasonics Corp. (Trenton, USA) within a sealed glass vial to prevent ethanol loss during the process. The prepared ethanolic solution was slowly introduced into preheated deionized water (final volume 10 mL) maintained at the same temperature, under constant magnetic stirring for 45 min [31]. Unlike conventional evaporation-based methods, ethanol was retained within the system to function as a permeability promoter, contributing to the vesicles’ deformability and improved membrane permeability [13]. The final ethanol concentration in the transethosomal formulations was maintained between 25–50% v/v. To further refine the dispersion and minimize particle size, the resultant transethosomal suspension was subjected to sonication for 20 min at 25 °C (Ultra Sonicator, model LC 60/H, Elma, Singen, Germany). The optimized formulations were kept under refrigeration (4 °C) until further characterization and evaluation [32, 33].

### In-vitro evaluations of MOD-entrapped transethosomes

#### Assessment of entrapment efficiency

Encapsulation efficiency (EE%) was computed by quantifying the unbound drug concentration present in the continuous medium surrounding the transethosomal formulation. An indirect method was employed, whereby 1 mL of the synthesized dispersion underwent ultracentrifugation at 20,000 rpm for 60 min employing a cooled centrifuge set at 4 °C (Sigma 3K30, Germany). This process ensured complete separation of vesicular structures from the external aqueous phase [34]. The resulting clear supernatant, which contained the un-encapsulated drug fraction, was carefully withdrawn for further analysis. The Shimadzu UV-1601 PC unit (Kyoto, Japan) was utilized for spectrophotometric analysis to determine the free drug concentration in the supernatant at its maximum absorption wavelength (λmax = 230 nm). A validated calibration plot was established over a suitable concentration range, exhibiting excellent linearity (R^2^ = 0.9991). Prior to measurement, the supernatant samples were suitably diluted to ensure linearity in the concentration-absorbance plot. Finally, EE% was estimated as expressed in the following relation [35, 36]:

1 $$\text{EE \%}=\frac{(\text{Total amount of MOD}-\text{Unentrapped MOD})}{\text{Total amount of MOD}}\text{ x }100$$

#### Assessment of size dispersion and zeta potential

To evaluate the colloidal characteristics of the prepared MOD-loaded transethosomes, ZP, PS and PDI were systematically clarified. For each measurement, a 50 µL sample (accurately measured) from the fresh dispersion was diluted 100-fold with deionized water, yielding a medium with appropriate light scattering properties for instrumental analysis. Prior to analysis, the diluted samples were briefly mixed via vortexing to ensure vesicle homogeneity and prevent agglomeration. Dynamic Light Scattering (DLS) was subsequently performed on the Malvern Zetasizer Nano ZS (Worcestershire, UK). The instrument utilized 173° non-invasive backscatter optics, and all measurements took place at 25 °C [37, 38].

### Design-expert^®^-driven statistical optimization

The formulation was systematically designed and optimized utilizing Design-Expert^®^ software to produce a nanosystem with desirable characteristics. The optimization strategy aimed to achieve maximum EE% and high ZP to ensure vesicle stability, while maintaining PS strictly within the nano-metric range to ensure effective delivery. Statistical evaluation of the experimental design was conducted through analysis of variance (ANOVA), providing the means to quantify the impact of the input parameters relative to the dependent variables [39]. We subsequently implemented a numerical optimization strategy utilizing the desirability function as the main metric, where higher values (close to 1) indicated better compliance with the optimization goals. The optimal preparation, identified by the peak desirability value, was chosen for extensive characterization [40, 41].

### Comprehensive physicochemical profiling of the optimized formulation

#### Morphological elucidation by TEM imaging

We visualized the shape of the optimized formulation using the JEOL JEM-1230 TEM instrument (Tokyo, Japan), set to an acceleration potential of 80 kV. For sample preparation, the formulation underwent adequate dilution using deionized water for the purpose of achieving un-clustered particles. A minute drop of the dispersion was cautiously mounted onto copper grids supported by a carbon film to provide adequate support for imaging. To improve image contrast and highlight the surface outline, Negative staining was performed on the grids, utilizing a 2% (w/v) solution of phosphotungstic acid, followed by gentle air-drying at ambient conditions [42–44].

#### FTIR-assisted functional group analysis

FTIR spectroscopy served as the primary verification method to assess structural compatibility between MOD and the excipients, and concurrently to confirm successful drug encapsulation within the final transethosomes system. The spectral profiles of the pure drug and individual components (such as PC and cholesterol) were recorded alongside that of the optimized formula utilizing a Bruker FTIR spectrophotometer (Model 22, Coventry, UK). Following extensive drying (to ensure the absence of water-induced spectral artifacts), the samples were co-compressed with transparent KBr powder to yield the analytical pellet. The spectral measurements were conducted under thermostatically controlled conditions (25 °C), spanning the wavenumber region of 4000–500 cm⁻^1^. The comparative evaluation of characteristic absorption bands allowed detection of any shifts, disappearance, or emergence of new peaks, which serve as indicators of molecular interactions or structural modifications within the vesicular matrix [28, 45].

#### DSC analysis

We first lyophilized the optimized formula to yield a stable, desiccated powder. The process utilized the Savant Instruments Novalyphe-NL 500 unit (NY, USA). The procedure involved two sequential stages: an initial freezing step at –20 °C, Subsequent moisture removal was performed over a 24-h period, utilizing a cryogenic temperature (−45 °C) and sub-atmospheric pressure [12]. To evaluate the thermal transitions of the components, Differential Scanning Calorimetry (DSC) was subsequently conducted using the PerkinElmer DSC7 unit (Waltham, MA, USA). Approximately 2 mg of the individual materials (pure MOD, PC, cholesterol) and the freeze-dried optimized formula were sealed in aluminum pans and thermally ramped to 350 °C at a rate of 10 °C/min under a nitrogen purge [46].

#### In-vitro release profiling and kinetic modeling

To investigate the transethosomes’ influence on drug release kinetics, the in-vitro dissolution profile of the optimized MOD-loaded transethosomes was benchmarked against a conventional drug suspension. The study relied on the dialysis bag diffusion method, utilizing a molecular weight cut-off of 12,000–14,000 Da. Prior to use, the membranes were soaked overnight in release medium to ensure proper hydration [47]. An accurately measured dose equivalent to 1 mg of MOD, either from the optimized formulation or the drug suspension, was enclosed within the dialysis membrane and immersed in 50 mL release medium (20% v/v ethanoic phosphate-buffered saline, pH 6.4) maintained at 37 ± 0.5 °C under continuous stirring at 100 rpm to closely simulate the physiological conditions of nasal fluid, ensuring the release profile reflects the environment at the site of administration [48, 49]. Over the 8-h study, we removed 3 mL samples from the receptor medium at set intervals. This volume was instantly replaced with fresh pre-warmed buffer to ensure constancy of volume and preservation of the concentration gradient [31]. The amount of released MOD was assayed via UV–Vis spectroscopy at its λmax and the cumulative percent release was graphed against time. The resulting dissolution profiles were subsequently subjected to fitting against four models (zero-order, first-order, Higuchi, and Korsmeyer–Peppas) to determine the governing release mechanism. The model that provided the highest correlation coefficient (R^2^) was selected as the best fit for the observed kinetics [50, 51].

#### pH measurement

A calibrated Jenway model-3505 pH meter (Staffordshire, UK) was used to determine the pH of the optimum formula to ensure its suitability for intranasal applications. The optimum formula was relocated to a clean beaker and thermostatically conditioned at ambient temperature before analysis. To guarantee the reliability of the readings, every measurement was repeated three times [12, 52].

#### Stability assessment

The optimized formulation was stored at 5 ± 3 °C for a period of 3 months in tightly sealed glass containers to evaluate its physicochemical stability. Following this refrigerated period, the product was visually inspected for any indication of vesicle aggregation, precipitation, or other physical deterioration. To determine the presence of significant variance induced by storage, the core physicochemical metrics (EE%, PS, and ZP) were re-evaluated. The post-storage data was then benchmarked against the original values using one-way analysis of variance (ANOVA) [53, 54]. In parallel, we replicated the drug release study under the initial conditions to assess the similarity of the release kinetics before and after refrigeration. This similarity was quantified using the f_2_ metric, which is determined by the equation below [55, 56]:

2 $${\mathrm{f}}_{2}=50.\mathrm{log}\{[1+\left(\frac{1}{\mathrm{n}}\right){\sum }_{\mathrm{t}=1}^{\mathrm{n}}{\left({\mathrm{R}}_{\mathrm{t}}-{\mathrm{T}}_{\mathrm{t}}\right)}^{2}{]}^{-0.5}.100$$

where R_t_ and T_t_ represent the percent cumulative drug dissolution values recorded at each sampling time, corresponding to the initial and final storage time points, respectively. An f_2_ value exceeding 50 was considered indicative of no significant difference between the two release profiles, thus confirming the preservation of the formulation’s release characteristics [57].

### Ex-vivo study

#### Ethics approval

Ethical standards and proper study reporting were guaranteed by performing all experimental procedures in strict adherence to the ARRIVE guidelines. The entire animal study protocol was first formally reviewed and sanctioned by the Research Ethics Committee of the National Centre for Radiation Research and Technology (REC-NCRRT, Egyptian Atomic Energy Authority), assigned Approval No.: F/17A/25.

#### Isolation of sheep nasal mucosa

Fresh heads of sheep (approximately 1–1.5 years old, weighing 55–60 kg) were sourced from a licensed local slaughterhouse in Cairo, Egypt. To preserve tissue viability for subsequent ex-vivo investigations, nasal mucosa was excised within 10 min of sacrifice [12]. A longitudinal incision was performed along the lateral wall of the nasal cavity, ensuring full exposure without septal injury [12]. The mucosal layer was then carefully separated, gently rinsed, and instantaneously dipped in ice-cold Ringer’s solution to maintain its structural and functional integrity [47].

#### Ex-vivo permeation studies

For the permeation study, the excised sheep nasal mucosa was carefully situated to divide the donor and receptor compartments of the Franz apparatus. The donor chamber was filled with 1 mL of either the optimized MOD-loaded transethosomal formulation or a plain MOD suspension (1 mg/mL). The receptor chamber was charged with 25 mL of the 20% v/v ethanoic PBS (pH 6.4). Throughout the experiment, the medium was maintained at 37 ± 0.5 °C and stirred constantly at 100 rpm to match the physiological environment [12]. Over the designated time points within 10 h, 2 mL samples were extracted from the receptor medium. The withdrawn volume was instantly offset by adding 2 mL of fresh ethanoic buffer to sustain the necessary sink conditions [58]. Prior to spectrophotometric assay, all samples were passed through a 0.22 nylon filter. The cumulative amount of drug transported per unit area (Q) was then graphed against the time elapsed. Permeation kinetics were quantified by determining the maximum flux at 10 h (Jmax) and the enhancement ratio (ER). These metrics, essential for benchmarking against the control suspension, were calculated using the following formulas [36]:

3 $${\mathrm{J}}_{\mathrm{max}} =\frac{\text{Amount of drug permeated}}{\text{Time X Area}}$$

4 $$\mathrm{ER}=\frac{\text{Jmax of optimum formula}}{\text{Jmax of control}}$$

### In vivo histopathological evaluation

Histopathological analysis was employed to verify the absence of adverse local effects and assess the biocompatibility profile of the components within the final product. Six Swiss mice were randomly allocated into two groups: a control group (Group I) and a treated group (Group II), which received the optimized preparation. The formulation was administered intranasally three times per day for seven consecutive days. The nasal mucosa was carefully excised and retrieved for histological examination. This was done after the experimental subjects were euthanized using a ketamine/xylene protocol at the conclusion of the treatment period [59].

### Radio-kinetic analysis

#### Radiolabeling of MOD

Radiolabeling of MOD was performed using freshly eluted sodium pertechnetate [^99m^Tc]NaTcO₄ under reductive conditions with sodium dithionite (Na₂S₂O₄) as the reducing agent. To optimize the labeling process, the effects of multiple parameters were systematically examined, including drug amount, amount of Na₂S₂O₄, incubation time, and temperature. To ensure rigor, all tests were replicated three times. The outcomes are summarized using the mean ± SD. Different amounts of MOD were used (1 to 14 mg) and dissolved in 1 mL of solvent containing equal volumes of ethanol and DMSO. After that, an aqueous solution of dithionite was added (1–8 mg). Exactly 100 μL (8.9 MBq) of [^99m^Tc]Tc was added. The components were mixed vigorously using a magnetic stirrer. The resulting product was then tested at five specific temperature settings (35, 40, 60, 80 and 100 °C) and five different incubation periods (ranging from 5 to 60 min).

#### Paper chromatographic analysis

Ascending paper chromatography (PC) was employed using Whatman No. 1 paper strips (13 cm × 1 cm) in each labeling trial [60]. Approximately 2 cm from the lower edge, small aliquots of the radiolabeled reaction mixture were spotted onto the origin line. When acetone was applied as the mobile phase, unbound [^99m^Tc]Tc migrated with the solvent front (R_f_ = 0.8–1.0), whereas both [^99m^Tc]Tc-MOD and the reduced/hydrolyzed technetium (R/H)-[^99m^Tc]NaTcO_4_ remained at the baseline (R_f_ = 0). To further discriminate reduced/hydrolyzed impurities, an additional mobile phase consisting of ethanol: water: ammonium hydroxide (2:5:1, v/v/v) was used. Under these conditions, the (R/H)-[^99m^Tc]NaTcO_4_ fraction persisted at the origin (R_f_ = 0), while free [^99m^Tc]Tc and [^99m^Tc]Tc-MOD migrated with the solvent front (R_f_ = 0.9–1.0). The percentage of the RCY of [^99m^Tc]Tc-MOD complex was determined using the following equations:

5 $$\text{Free }{[}^{99\mathrm{m}}\mathrm{Tc}]{\mathrm{TcO}}_{{4}^{-}}=[\text{Radioactivity at }{\mathrm{R}}_{\mathrm{f}}=0.8-1.0(\mathrm{acetone})/\text{Total radioactivity}]\times 100$$

6 $$\mathrm{R}/\mathrm{H})-{[}^{99\mathrm{m}}\mathrm{Tc}]{\mathrm{NaTcO}}_{4}=[\text{Radioactivity at }{\mathrm{R}}_{\text{f }}=0.0 (\mathrm{mixture})/\text{Total radioactivity}] \times 100$$

7 $$\mathrm{\%}{[}^{99\mathrm{m}}\mathrm{Tc}]\mathrm{Tc}-\mathrm{MOD}=100-({\% free }{[}^{99\mathrm{m}}\mathrm{Tc}]{\mathrm{TcO}}_{{4}^{-}}+{\% }(\mathrm{R}/\mathrm{H})- {[}^{99\mathrm{m}}\mathrm{Tc}]{\mathrm{NaTcO}}_{4})$$

#### In-vitro serum stability of radiolabeled compound

The in-vitro serum stability of [^99m^Tc]Tc-MOD transethosomes was evaluated for 5 h using human serum at 37 °C (compound: serum ratio of 1:8). The Sesa Uniscaller well-type gamma counter was deployed for the assessment of radiochemical yield. Samples (2 µL, n = 3) were drawn from the reaction mixture at pre-determined times (0.5, 1, 2, 3, 4, and 5 h post-incubation) and subjected to the previously described chromatographic procedure [61, 62].

#### Preparation of [^99m^Tc]Tc-MOD transethosomes

The radiolabeled MOD-loaded transethosomal system [^99m^Tc]Tc-MOD transethosomes was fabricated following the same ethanol injection technique previously outlined for non-labeled formulations (Sect. "Development of MOD-entrapped transethosomes"). In this procedure, purified [^99m^Tc]Tc-MOD was incorporated as the active component, replacing the unlabeled drug. This ensured that the labeling step was seamlessly integrated into the vesicle construction process, allowing the nanocarrier to retain its structural and functional characteristics while enabling subsequent radio-kinetic tracking.

#### EE%, PS and ZP after labeling

We quantified the EE% following a centrifugation step (DAIHAN Scientific, Korea) performed at 20,000 rpm for one hour at ambient temperature. Using a UV/Vis spectrophotometer (UK), the MOD that persisted in the supernatant was measured spectrophotometrically. Particle size (PS) and zeta potential (ZP) were measured as previously described using a Malvern Zetasizer Nano ZS (Worcestershire, UK).

#### In-vivo behavior of [^99m^Tc]Tc-MOD

A total of forty-five healthy male Swiss Albino mice, weighing between 20–30 g, were randomly allocated into three experimental groups (I, II, and III; n = 15 per group, subdivided into three animals per time point). Swiss mice were selected as animal models in the current study because they are small and manageable for intranasal administration during in-vivo studies [63, 64]. Animals were housed individually in metabolic cages with controlled access to food and water at scheduled intervals. Based on previous reports, body weight distribution was estimated as 10% muscle, 7% blood, and 40% bone mass [65]. Group I received an intravenous injection of [^99m^Tc]Tc-MOD solution (Vs); Group II was intranasally administered the same solution (Ns), while Group III was intranasally treated with [^99m^Tc]Tc-MOD transethosomes (NTE). Each mouse was administered approximately 20 μL of the radiolabeled preparation containing 0.285 μg MOD (200–400 KBq of [^99m^Tc]Tc) [25]. Post-administration, animals were sacrificed at predetermined time points (5, 15, 30, 60, and 120 min). Anesthesia was achieved by intraperitoneal injection of 0.1 mL of a freshly prepared stock containing xylazine (2.5 mg/mL) and ketamine HCl (25 mg/mL) [66], followed by euthanasia via cervical dislocation. For each administration route and time interval, the brain/blood ratio (B/B) was calculated. Animals were positioned appropriately for nasal dosing, organs were excised, rinsed twice with sterile saline, blotted, and accurately weighed. Brains were isolated, frozen, and recorded separately. Radioactivity within each organ was quantified using a γ-scintillation counter equipped with an NaI (Tl) detector. The percentage of injected dose per gram of tissue (%ID/g ± SD) was calculated for each sample according to the following equation [67, 68]:

8 $$\mathrm{\%ID}=[\text{Activity of tissue }(\mathrm{organ})/\text{Total injected activity}\times \text{weight of tissue }(\mathrm{organ})]\times 100$$

#### Pharmacokinetic study

Pharmacokinetic assessments were carried out using Kinetica™ 2000 software (version 3, InnaPhase Corporation), applying the linear trapezoidal method for curve fitting. The software generated key parameters, including the maximum brain concentration of radiolabeled MOD (C_max_), the time required to achieve peak concentration (T_max_), and the area under the concentration–time curve calculated from 0 to 120 min (AUC_0–120_) as well as extrapolated to infinity (AUC_0–∞_). The ability of the formula to target the brain through the nasal route has been assessed using drug targeting efficiency (DTE) and direct transport percentage from the nose to the brain (DTP) [69]. DTE represents the average partitioning ratio of [^99m^Tc]Tc-MOD between the brain and the blood for each the solution and the formula separately, while DTP represents the percentage of [^99m^Tc]Tc-MOD delivered to the brain via the direct transport route. The degree of drug targeting to the brain after intranasal administration can be evaluated by DTI, which can be described as the ratio of the value of AUC brain/AUC blood following nasal administration to that the intravenous injection. The higher the DTI is, the better the drug targeting to the brain can be expected after nasal administration [70]. Brain targeting efficiency, that is, DTE% and DTP%, which represents the time average partitioning ratio, are calculated as follows:

9 $$\text{DTE \%}=[\text{AUC }(0-120)\text{ brain}/\text{AUC }(0-120)\mathrm{blood}]\times 100$$

where AUC (0–120) is for transethosomes and solution following nasal administration.

To estimate DTP%, the AUC of brain fraction (AUC_bf_) was calculated first after the intranasal distribution:

10 $${\mathrm{AUC}}_{\mathrm{bf}}=[{\mathrm{AUC}}_{0-\mathrm{last}}\text{ Vs brain}/{\mathrm{AUC}}_{0-\mathrm{last}}\text{ Vs blood}]\times {\mathrm{AUC}}_{0-\mathrm{last}}\mathrm{N}$$

where AUC_(0-last)_ N is AUC_(0–120)_ for blood after intranasal delivery, and AUC_(0-last)_ Vs is AUC_(0–120)_ for brain after intravenous delivery.

11 $$\mathrm{DTP\%}=[{\mathrm{AUC}}_{0-\mathrm{last}}\mathrm{NB}-{\mathrm{AUC}}_{\mathrm{bf}}/{\mathrm{AUC}}_{0-\mathrm{last}}\mathrm{NB}]\times 100$$

where AUC_(0-last)_NB is AUC_(0–120)_ for the brain following intranasal distribution (nasal solution and nasal transethosomes), and AUC_bf_ was previously determined using Eq. (10).

12 $$\mathrm{DTI}=[\text{AUC brain}/\text{AUC blood }(\mathrm{nasal})]/[\text{AUC brain}/\text{AUC blood }(\mathrm{intravenous})]\times 100$$

whereas AUC brain/AUC blood (nasal) is AUC_(0–120)_ for nasal administration (nasal solution and nasal transethosomes), AUC brain/AUC blood (intravenous) is AUC_(0–120)_ for intravenous injections.

The percentage of relative bioavailability was calculated according to the following equation:

13 $${\% Relative availability}=[{\mathrm{AUC}}_{0-\infty }\mathrm{NTs}/{\mathrm{AUC}}_{0-\infty }\mathrm{Ns}]\times 100$$

Transethosomes are represented by AUC_(0-∞)_NTs, while nasal solution is represented by AUC_(0-∞)_Ns [71].

### Statistical evaluation

The dataset generated from the factorial design experiments was statistically interpreted using Design-Expert^®^ software (Stat-Ease Inc., Minneapolis, MN, USA), while additional statistical analyses were conducted with SPSS^®^ software (version 7.5.1, SPSS Inc., Chicago, IL, USA). To evaluate the significance of formulation variables and compare outcomes among different groups, a one-way analysis of variance (ANOVA) was applied, followed by the least significant difference (LSD) post hoc test to identify pairwise variations. A confidence level of 95% (*p* < 0.05) was adopted as the threshold for statistical significance. All measurements were performed in triplicate (n = 3), and the experimental data were expressed as mean values accompanied by their respective standard deviations to ensure reliability and reproducibility of the findings [38, 42, 46].

## Results and discussion

### Analysis of factorial design

The experimental outcomes were statistically analyzed using Design-Expert^®^ software. The selected design confirmed excellent model adequacy, with high coefficients of determination (R^2^) and minimal differences between adjusted and predicted R^2^ values (< 0.2), reflecting strong predictive accuracy. Adequate precision values for all responses exceeded the threshold of 4 (Table 3), validating the robustness of the models and their reliability in navigating the design space [72, 73].

**Table 3:** Statistical interpretation of the studied responses

Response	R^2^	Adjusted R^2^	Predicated R^2^	Adequate precision	Significant factors
**EE %**	0.9855	0.9746	0.9419	23.86	A,C
**PS (nm)**	0.8534	0.7434	0.7136	7.55	A,B
**ZP (mV)**	0.9222	0.8638	0.7886	11.53	A,B,C

*EE %* percent entrapment efficiency, *PS* Particle size, *ZP* Zeta potential

#### Statistical evaluation of EE%

**Fig. 1:**
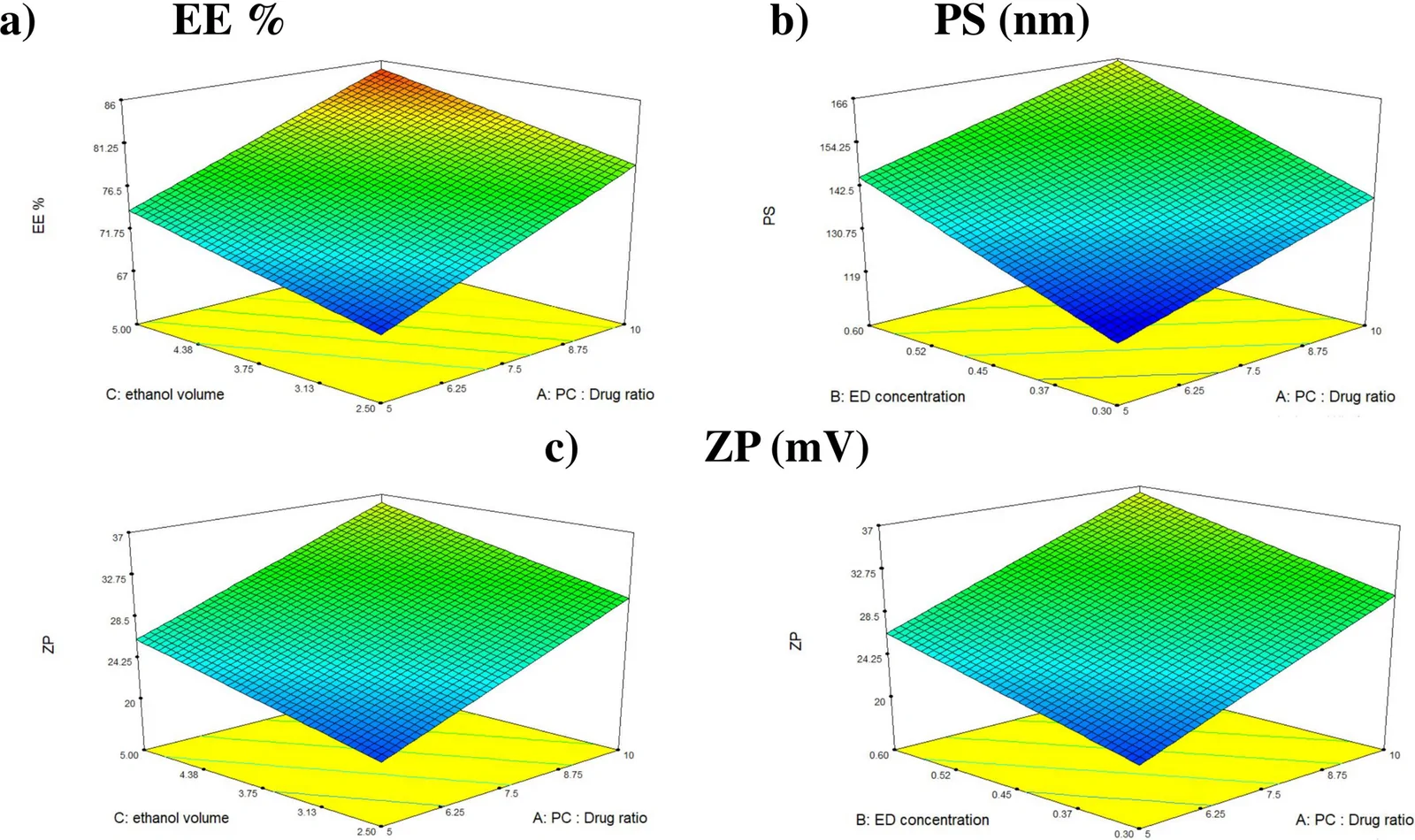
3D-Response plots for the effect of PC: Drug ratio (Factor A), ED concentration (Factor B) and Ethanol volume (Factor C) on (**a**) EE %, (**b**) PS and (**c**) ZP

In the present study, MOD-loaded transethosomes exhibited EE% values ranging from 67.15 ± 1.08% to 85.87 ± 5.15%, as shown in Table 2. Statistical analysis using ANOVA confirmed the significance of the model (*p* < 0.05), with phosphatidylcholine-to-drug ratio (factor A) and ethanol volume (factor C) exerting significant positive effects on EE%, while edge activator concentration (factor B) showed only a minor non-significant influence. Figure 1a illustrates 3D-Response plot for the effect of significant factors. The regression model describing the relationship between the variables and EE% is expressed as:

14 $$\mathrm{EE\%}=76.48+5.55\mathrm{A}+0.63\mathrm{B}+3.01\mathrm{C}.$$

The enhancement in entrapment efficiency (EE %) observed with increasing phosphatidylcholine (PC) concentration can be explained by several structural and physicochemical contributions. PC possesses inherently low aqueous solubility, which enriches the lipophilic environment of the formulation and strengthens the affinity between the drug and the lipid matrix [35]. A higher PC: drug ratio supplies a greater number of lipid bilayers, enabling more effective encapsulation of MOD molecules within the vesicular core [74]. The abundant bilayer material also imparts rigidity to the vesicle walls, decreases their fluidity, and increases the overall viscosity of the dispersion. These changes collectively minimize drug diffusion out of the vesicles, thereby maintaining higher levels of drug retention and prolonging stability [75]. Ethanol volume (factor C) also played a significant role in improving EE%. Ethanol acts as both a solvent and a structural modulator: it enhances solubilization of the lipid components as well as the drug itself, while simultaneously contributing to the proper self-assembly of the vesicular bilayers. Through its ability to form hydrogen bonds with the carbonyl and carboxylic groups of MOD, as well as with the polar head groups of phospholipids and cholesterol, ethanol promotes a stable and coherent vesicle network [76]. This synergistic effect ensures not only greater drug incorporation within the bilayer but also improved structural organization of the transethosomal vesicles.

#### Statistical evaluation of PS

In this study, MOD-loaded transethosomes demonstrated nano-metric sizes, ranging from 121.75 ± 1.20 nm to 180.30 ± 1.70 nm (Table 2), ensuring suitability for systemic circulation and effective brain targeting. Statistical analysis confirmed the overall model significance (*p* < 0.05), with both the phosphatidylcholine-to-drug ratio (factor A) and edge activator concentration (factor B) exerting significant positive influences on PS, while ethanol volume (factor C) had a relatively minor non-significant effect. Figure 1b illustrates 3D-Response plot for the effect of significant factors The relationship between variables and particle size was described by the following coded equation:

15 $$\mathrm{PS}=142.44+10.11\mathrm{A}+12.90\mathrm{B}+3.08\mathrm{C}.$$

The observed enlargement of vesicle size with increasing phosphatidylcholine (PC) concentration can be attributed to its structural role as the primary backbone of the lipid bilayer. A higher PC: drug ratio supplies additional lipid molecules that assemble into more extensive bilayer structures, thereby producing vesicles with thicker membranes and an expanded aqueous core for accommodating MOD [77]. The bulkier architecture of PC, combined with its amphiphilic nature, encourages the formation of paucilamellar or multilamellar vesicles rather than smaller unilamellar ones, which inherently increases particle size. Moreover, the abundant bilayer material reinforces the rigidity of the vesicle walls, reduces bilayer fluidity, and raises the viscosity of the dispersion medium. These physicochemical changes not only minimize drug leakage from the vesicular core but also favor the development of larger, more stable transethosomes. Similarly, the positive correlation between edge activator (EA) concentration and particle size was evident. At higher EA levels, the system yielded larger vesicles, which can be attributed to the physicochemical properties of the surfactant employed. Hydrophilic surfactants such as Cremophor^®^ RH 40 (HLB ≈ 14–16) tend to increase vesicle size due to their high surface free energy, which interferes with tight lipid packing during vesicle self-assembly [17]. Additionally, incorporation of more EA molecules within the lipid bilayer can lead to steric hindrance and partial disruption of lipid alignment, thereby enlarging the vesicular structure [78]. The ability of surfactants to reduce interfacial tension while simultaneously promoting bilayer expansion explains their role in particle growth.

#### Statistical evaluation of PDI

PDI values closer to zero represent a narrow size distribution and greater homogeneity, which are typically associated with improved stability. In this study, the measured PDI values of the prepared transethosomal formulations fell within the acceptable nanocarrier threshold (< 0.5), ranging from 0.16 ± 0.01 to 0.27 ± 0.03 (Table 2) [79]. These results confirm that the vesicles across all batches were consistently uniform, with no evidence of aggregation or heterogeneity. Statistical analysis (ANOVA) demonstrated that none of the investigated formulation variables had a statistically significant effect on PDI (*p* > 0.05). Consequently, PDI was not included as a determinant parameter during the optimization process, given its consistently favorable values across all formulations.

#### Statistical evaluation of ZP

Systems with ZP values exceeding ± 30 mV are generally considered stable, as the strong repulsive forces effectively minimize vesicle aggregation [54]. In the present study, the prepared MOD-loaded transethosomes exhibited ZP values ranging from −19.05 ± 1.63to −42.60 ± 1.27 mV (Table 2), indicating that all formulations possessed a sufficiently high negative charge to ensure good colloidal stability. Statistical evaluation confirmed that all three formulation variables exerted a significant positive effect on ZP (*p* < 0.05), which is illustrated as 3D-Response plot in Fig. 1C. ZP is mathematically represented by the coded equation:

16 $$\mathrm{ZP}=28.36+4.93\mathrm{A}+3.06\mathrm{B}+2.81\mathrm{C}.$$

The influence of phosphatidylcholine (PC) can be explained by its amphiphilic nature and bulk bilayer-forming structure. Increasing PC content enriches the vesicular bilayer, leading to greater surface exposure of negatively charged phosphate groups, thereby amplifying electrostatic repulsion. Ethanol, in turn, further contributed to the negative surface potential by enhancing the ionization of phospholipid head groups and increasing membrane fluidity, which facilitates the outward orientation of anionic phosphate moieties [80]. Additionally, the edge activator played a pivotal role in improving stability. By adsorbing at the vesicle interface, edge activator molecules provided steric hindrance that prevented vesicles from approaching each other closely, thereby reducing aggregation. This steric stabilization complemented the electrostatic repulsion and further reinforced colloidal stability [17].

### Design-expert^®^-driven statistical optimization

Optimization of the formulation parameters was performed using the numerical desirability function approach incorporated in Design-Expert^®^ software. This statistical tool enabled systematic evaluation of multiple factors to determine the most favourable composition that simultaneously satisfied the predefined criteria for entrapment efficiency (EE%), particle size (PS), and zeta potential (ZP). The optimization strategy was designed to maximize EE% and ZP while maintaining PS within the nanoscale range. The program generated several candidate formulations with desirability values ranging between 0 and 1, where higher values indicated superior compliance with the targeted responses. Among these, the optimum formulation (F5) was obtained at factor levels of (A) phosphatidylcholine-to-drug ratio = 10:1, (B) edge activator concentration = 0.6% w/v, and (C) ethanol volume = 5 mL, which yielded the highest overall desirability value of 0.919. This optimized composition was then selected for experimental validation and subsequent investigations (Table 4) [81].

**Table 4:** Characterization of the optimum formula

Response	Y1	Y2	Y4
	EE %	PS (nm)	ZP (mV)
**Observed value**	85.87	180.30	−42.60
**Predicated value**	85.67	172.52	−41.15
**% Deviation (absolute)**	0.24	4.51	3.53

*EE %* percent entrapment efficiency, *PS* Particle size, *ZP* zeta potential

### Comprehensive physicochemical profiling of the optimized formulation

#### Morphological elucidation by TEM imaging

**Fig. 2:**
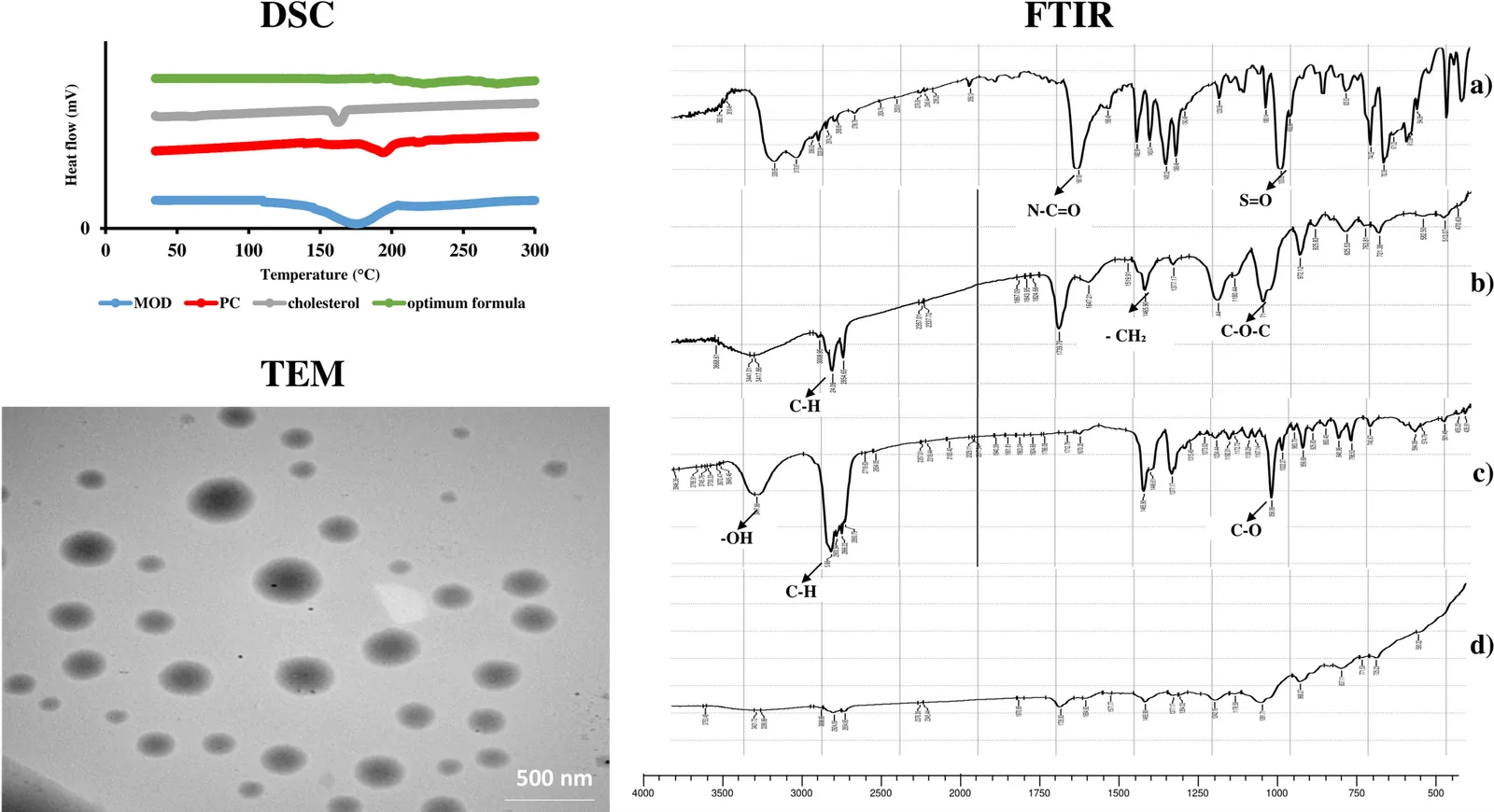
DSC thermograms (upper left) and FTIR spectra (right) of (**a**) pure MOD, (**b**) phosphatidylcholine (PC), (**c**) cholesterol, and (**d**) the optimized formulation, with labeled characteristic peaks confirming successful encapsulation. A TEM image of the optimized formulation is also presented (lower left)

Transmission electron microscopy (TEM) micrographs of the optimized MOD-loaded transethosomes (Fig. 2) revealed well-defined, spherical vesicles with a homogeneous size distribution, closely matching the particle size data obtained from Malvern Zetasizer. The absence of clustering, fusion, or irregular shapes confirmed the structural integrity and colloidal stability of the system, which can be attributed to the combined effect of electrostatic repulsion from the negative surface charge and steric stabilization provided by the formulation components [82, 83].

#### FTIR-assisted functional group analysis

Fourier-transform infrared (FTIR) spectroscopy was employed to assess potential interactions between MOD and the selected excipients in the optimized formulation (Fig. 2). The FTIR spectrum of pure phosphatidylcholine (PC) revealed its characteristic signals, including aliphatic C–H stretching vibrations near 2900 cm⁻^1^, ester C = O stretching around 1740 cm⁻^1^, methylene (–CH₂) scissoring of the phospholipid acyl chains at 1465 cm⁻^1^, and C–O–C stretching at approximately 1069 cm⁻^1^, all of which confirm its phospholipid backbone [36]. Cholesterol exhibited distinctive absorption bands at 3418 cm⁻^1^ (O–H stretching), 2800–3000 cm⁻^1^ (aliphatic C–H stretching), 1465 cm⁻^1^ (CH₂ scissoring), and 1378 cm⁻^1^ (CH₃ bending), along with fingerprint signals between 1050–1150 cm⁻^1^ attributed to C–O stretching of the hydroxyl group [84]. MOD displayed multiple characteristic peaks: amide carbonyl vibrations in the range of 1630–1680 cm⁻^1^, C–C stretching between 1650–1200 cm⁻^1^ and the sulfoxide (S = O) functional group at 1065–1030 cm⁻^1^ [85]. In the FTIR spectrum of the optimized transethosomal formulation, several distinct peaks of MOD appeared significantly reduced or completely masked, particularly within the carbonyl and sulfoxide regions [86]. These findings strongly support successful drug incorporation and molecular compatibility among the formulation components, providing further evidence of stable vesicle formation suitable for brain-targeted drug delivery [35].

#### DSC analysis

The DSC profiles of MOD, PC, cholesterol, and the freeze-dried optimized formulation are presented in Fig. 2. Pure modafinil exhibited a distinct melting transition at approximately 170 °C, confirming its crystalline nature [86]. PC displayed an endothermic signal near 165 °C, consistent with its intrinsic lipid phase behavior [38]. Cholesterol demonstrated a notable peak at 149 °C representing its melting point [87]. In contrast, the thermogram of the optimized formulation no longer showed the sharp melting peak of Modafinil (~ 170 °C), indicating that the drug was successfully incorporated within the vesicular matrix and exists in an amorphous or molecularly dispersed state. This disappearance confirms efficient entrapment.

#### In-vitro release profiling and kinetic modeling

**Fig. 3:**
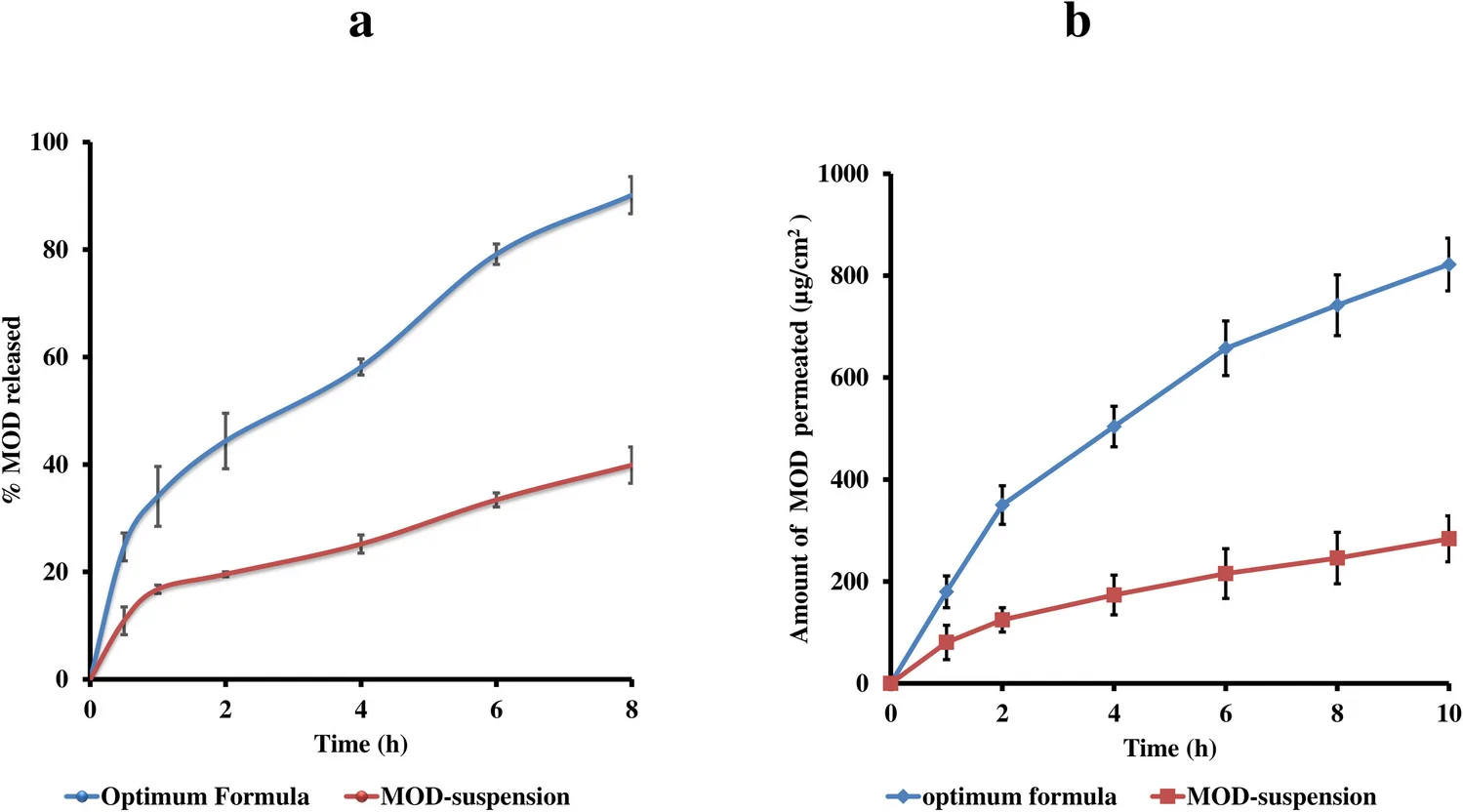
(**a**) In vitro release (**b**) Ex-vivo permeation profiles from optimum formula compared to that from MOD control, mean ± SD, *n* = 3

The in-vitro release profile of MOD from the optimized transethosomal formulation was compared with that of the plain drug suspension (Fig. 3a). The suspension displayed very limited dissolution after 8 h (39.9 ± 3.37%), releasing only a small fraction of MOD during the initial hours, reflecting the drug’s poor aqueous solubility. In contrast, the optimized transethosomal formulation exhibited a biphasic release pattern: an initial burst phase, attributed to un-encapsulated or surface-associated drug, was followed by a sustained and gradual release as MOD diffused from the lipid bilayers [88]. The initial burst is advantageous for modafinil because it facilitates rapid onset of action, which is particularly desirable for a wake-promoting agent intended to enhance alertness and cognitive performance. This is then followed by sustained release, ensuring prolonged drug availability and maintaining therapeutic brain levels without the need for frequent dosing. The highest release from transethosomes (90.1 ± 3.46%) was significantly greater than the suspension (p < 0.05), which can be attributed to the synergistic effects of the formulation components. Ethanol enhanced solubility and fluidized the lipid bilayer [35], while phosphatidylcholine and cholesterol contributed to vesicular rigidity and stability, ensuring sustained release. Importantly, the edge activator significantly facilitated drug liberation by imparting elasticity to the vesicle membrane, disrupting the tight packing of phospholipids, and thereby increasing permeability [17]. This structural flexibility not only improved the release rate but also supported deeper diffusion of MOD from the vesicular matrix. Kinetic modelling revealed a diffusion exponent (n) value of 0.224, which is ≤ 0.45, confirming that the optimized system follows a Fickian diffusion mechanism. This indicates that MOD release is primarily governed by diffusion from the lipid bilayers [37].

#### pH measurement

Maintaining a physiological pH is a crucial parameter for intranasal formulations, as even slight deviations can disrupt mucociliary clearance and provoke irritation of the nasal epithelium. The optimized MOD-loaded transethosomal formulation exhibited a pH of 5.68 ± 0.21, which falls within the normal pH range of nasal secretions (4.5–6.5) [12]. This close resemblance to the native environment suggests that the formulation is well-tolerated, minimizing the risk of mucosal irritation and thereby supporting its suitability for intranasal administration.

#### Stability assessment

The stability of the optimized formulation was systematically examined over a three-month period under refrigerated conditions (5 ± 3 °C). During storage, the system retained its original clear appearance with no evidence of precipitation, aggregation, or turbidity, indicating excellent physical stability. Key performance parameters including particle size, entrapment efficiency, and zeta potential remained statistically unchanged when compared to the freshly prepared batch (*p* > 0.05), confirming the robustness of the formulation (Table 5). Furthermore, the in-vitro release profile after storage closely matched the initial release behavior, with a calculated similarity factor of f₂ = 79.21, confirming preservation of its sustained-release characteristics [31, 89]. This high degree of stability can be attributed to the strong negative surface charge (ZP = –42.60 mV) that generates sufficient electrostatic repulsion to minimize vesicle–vesicle interactions and prevent aggregation [83]. The nanoscale dimension of the vesicles enhances these repulsive forces by increasing the exposed surface area. In addition, the presence of phosphatidylcholine and cholesterol provides steric hindrance and structural reinforcement of the bilayer, synergistically maintaining vesicle integrity.

**Table 5:** Analysis of the optimized formula after storage

Parameter	Fresh	Storage for 3 months at 4–8 °C	
		Value	Probability (*p*)*
**EE %**	85.87 ± 5.15	81.24 ± 4.11	0.425
**PS**	180.30 ± 1.70	187.10 ± 3.11	0.113
**ZP**	−42.60 ± 1.27	−39.75 ± 2.76	0.316

*EE %* percent entrapment efficiency, *PS* Particle size, *ZP* zeta potential

* One-way ANOVA analysis to compare between the freshly prepared and the stored optimum formula

### Ex-vivo permeation

Ex-vivo permeation studies across freshly excised sheep nasal mucosa demonstrated that the optimized nanosystem achieved significantly (*p* < 0.05) superior drug permeation compared with the corresponding plain suspension (Fig. 3b). After 10 h, the optimized formulation displayed a markedly higher cumulative permeated amount (Q10h = 821.96 ± 51.66 µg/cm^2^) and superior maximum flux (Jmax = 82.20 ± 5.17 µg/cm^2^/h), yielding an enhancement ratio of 2.9. This enhanced permeation is attributed to multiple formulation factors: the nanoscale size of the vesicles prolongs mucosal residence and facilitates diffusion across epithelial barriers [12]. Furthermore, ethanol acts as a penetration enhancer and bilayer fluidizer [42]. Moreover, the edge activator disrupts lipid packing within the epithelial membrane, increasing vesicle deformability [17]. Together, these components synergistically enhance drug transport across the nasal mucosa [90].

### In vivo histopathological evaluation

**Fig. 4:**
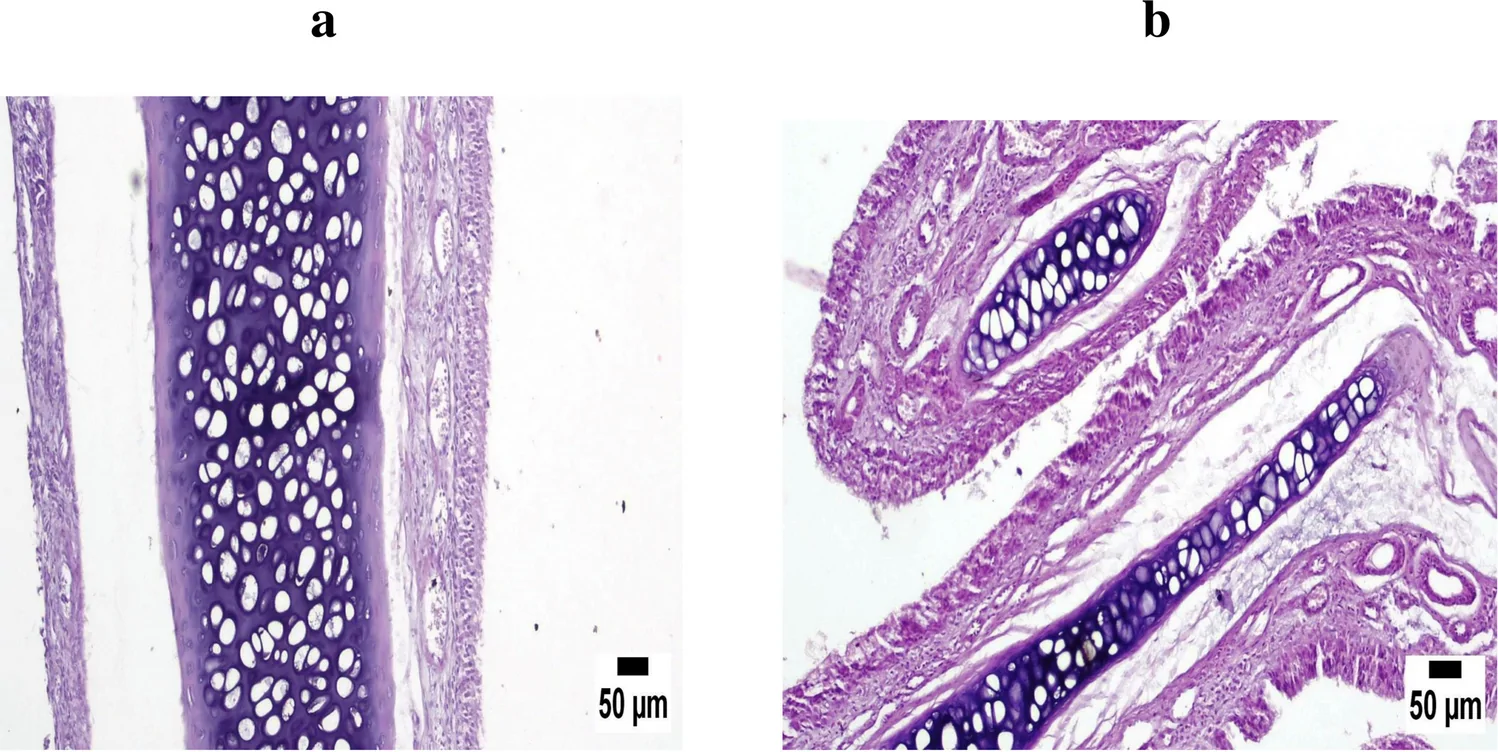
Histopathological examination of nasal mucosa following treatment: (**a**) control group displaying normal tissue architecture, and (**b**) optimized formul group demonstrating intact and healthy nasal mucosa

The histological observations demonstrated that the nasal tissues of animals treated with the optimized formula remained structurally intact and comparable to those of the control group (Fig. 4). No signs of irritation, epithelial disruption, or inflammatory response were detected. Collectively, these findings confirm that the optimum formula possess a favorable nasal mucosal safety profile, supporting their suitability for intranasal administration [12, 49].

### Radio-Kinetic analysis

#### Radiolabeling of modafinil

**Fig. 5:**
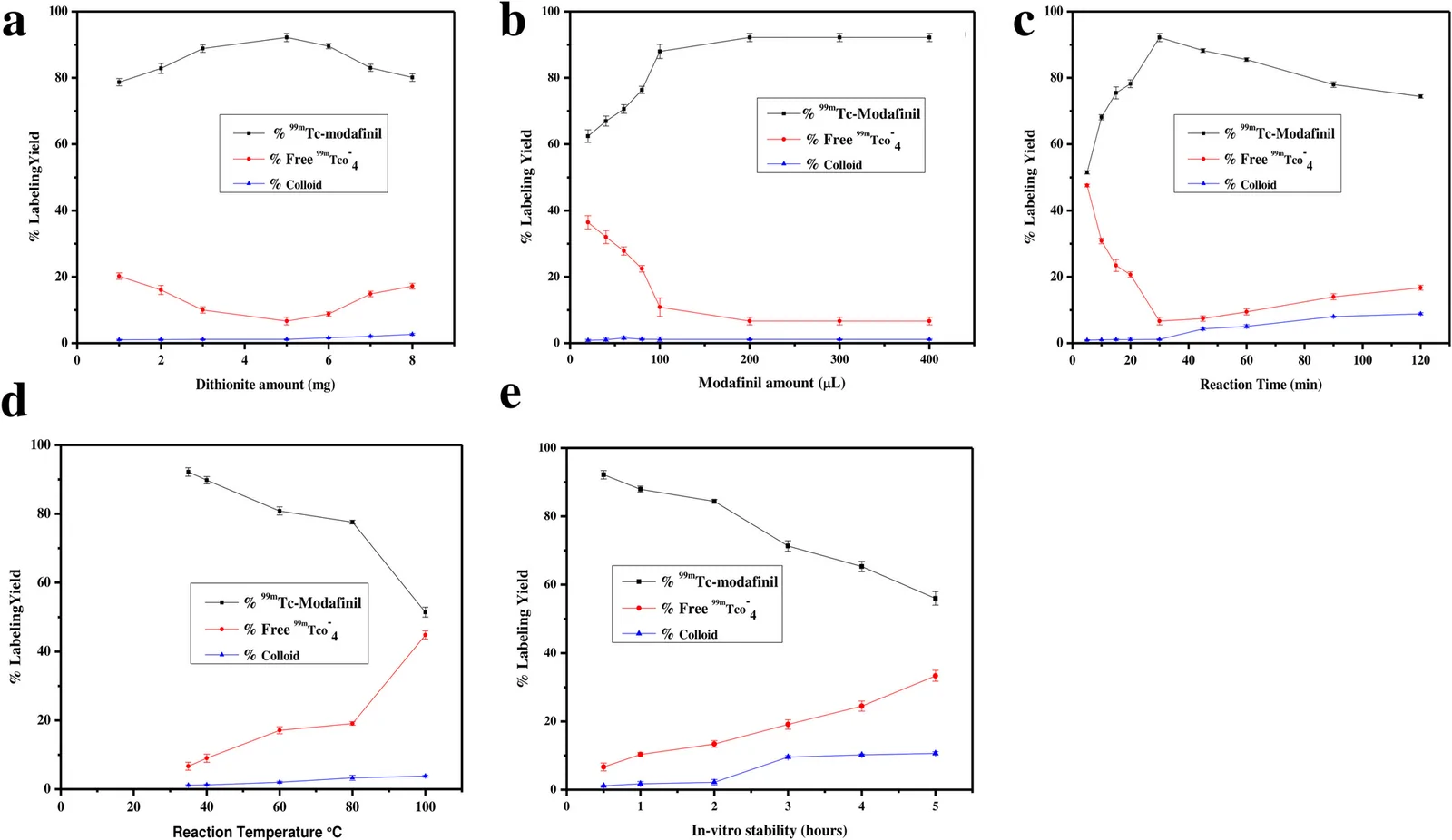
Changes in [^99m^Tc] Tc-modafinil radiochemical yield in relation to (**a**) sodium dithionite content (**b**) Modafinil quantity, (**c**) reaction time and (**d**) reaction temperature. In addition to (**e**) In-vitro serum stability

The %RCY of [^99m^Tc]Tc-MOD was significantly impacted by the Na₂S₂O₄ **(**Fig. 5a). 5 mg of Na₂S₂O₄ (*p* ≤ 0.05) produced the highest percentage of RCY (92.18% ± 1.2%). Below this threshold, the reduction of pertechnetate to the [^99m^Tc]Tc-complex could not be completed by Na₂S₂O₄. This accounts for the large amount of free pertechnetate present at low Na₂S₂O₄ concentrations. When Na₂S₂O₄ is used instead of stannous chloride, the absence of colloid from stannous oxidation, which produces stannic oxide, that interferes with the bio-distribution outcomes is an advantage [91, 92] from the processes. When the amount of Na₂S₂O₄ was increased above 5 mg, the percentage RCY decreased to 80.1% ± 1.1% at 8 mg of Na₂S₂O₄. This is due to the fact that a significant excess of dithionite will allow decreased [^99m^Tc]Tc to be somewhat re-oxidized, increasing the amount of free [^99m^Tc]TcO_4_^–^ [93]. One significant benefit of employing Na₂S₂O₄ as a reducing agent in the preparation of technetium-99 m radiopharmaceuticals is that, as observed, almost negligible colloidal species other than [^99m^Tc]TcO_2_ are obtained in the reaction. This helps to prevent colloidal stannic oxide interference for stannous chloride-prepared radiopharmaceuticals [91, 92]. The impact of MOD quantity on the % RCY is displayed in Fig. 5b. Using 200 µg of MOD per 20 µL of substrate, the labeling yield was 62.45% ± 1.88%. The limited amount of substrate, which was inadequate to form the complex with all reduced technetium, was the cause of the low RCY percentage. The percentage of RCY grew to its highest value of 92.18% ± 1.2% by increasing the amount of substrate to 2000 µg (200 µL). The percentage RCY was nearly the same when the amount of MOD was increased above the optimum value (200 µL); this could be because the substrate had reached its maximal saturation [48, 94]. The low RCY percentage (51.5% ± 0.5%) at the first 5 min, as seen in Fig. 5c, could be the result of not having enough time to develop [95]. The percentage RCY climbed to 92.18% ± 1.2% when the reaction time was extended to 30 min. However, when the reaction time is increased more than 30 min, the percentage of RCY significantly drops. The percentage RCY of [^99m^Tc]Tc-MOD declined as the reaction temperature elevated to higher levels, reaching 51.4% ± 1.4% at 100 °C. This could be explained by the [^99m^Tc]Tc-MOD complex’s thermal instability at high temperatures, as Fig. 5d illustrates. The highest yield of [^99m^Tc]Tc-MOD, 92.18% ± 1.2%, was obtained at temperature (35 °C) and the pH of the reaction was 6.

#### In-vitro serum stability of radiolabeled compound

During the initial phase of the drug development process, serum stability evaluation is crucial. Serum enzymes may cause the labeled material to break down or alter, which would impair its performance in-vivo. Therefore, the stability of [^99m^Tc]Tc-MOD transethosomes at (37 °C) with serum was assessed prior to conducting the biodistribution investigations. As shown in **(**Fig. 5e), the complex was stable for up to 2 h with % RCY 87.9% ± 0.9% or 84.4% ± 0.5%.

#### EE%, PS and ZP after labeling

The EE% following radiolabeling was 81.22 ± 2.07, with no statistically significant difference compared to the freshly prepared, unlabeled formulation. This indicates that the optimum formula maintains high stability, consistent with the stability previously demonstrated in serum. The pronounced stability can be attributed to the strong negative surface charges that prevent aggregation [49]. The nanoscale dimensions of the vesicles further enhance these repulsive forces by increasing the available surface area. Additionally, the presence of phosphatidylcholine and cholesterol contributes steric hindrance and structural reinforcement of the bilayer [21]. Furthermore, vesicle integrity after radiolabeling was evaluated by measuring particle size (PS) and zeta potential (ZP). The results showed that these parameters were non-significantly altered (p > 0.05) compared to the freshly prepared, unlabeled formulation, confirming that the radiolabeling process did not compromise vesicle integrity or stability.

#### Bio-distribution studies

**Fig. 6:**
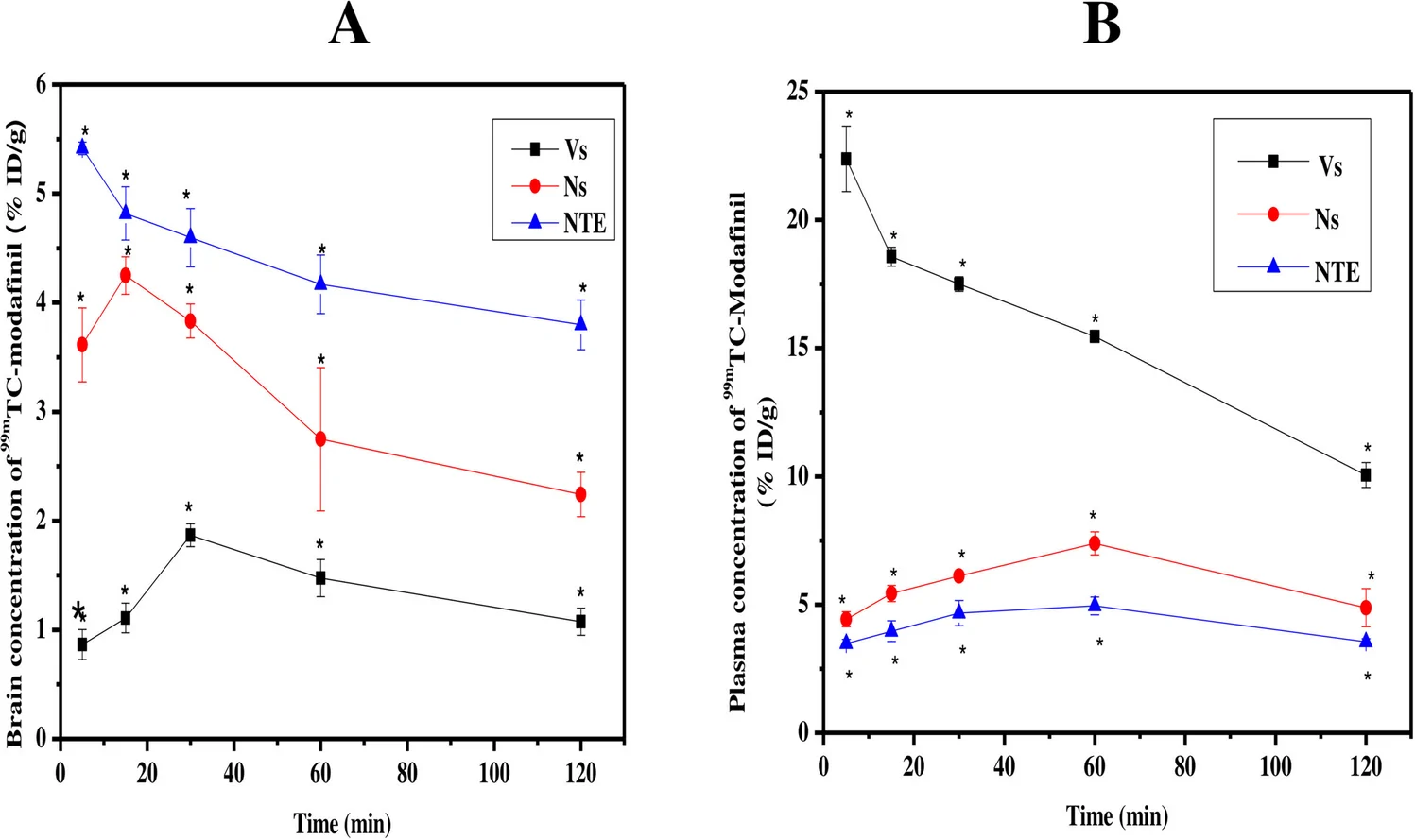
[^99m^Tc] Tc-modafinil uptake over time in; (**A**) Brain and (**B**) Plasma. * Suggests the existence of statistically significant variations in drug concentrations in the brain and plasma across various routes of delivery over time

**Fig. 7:**
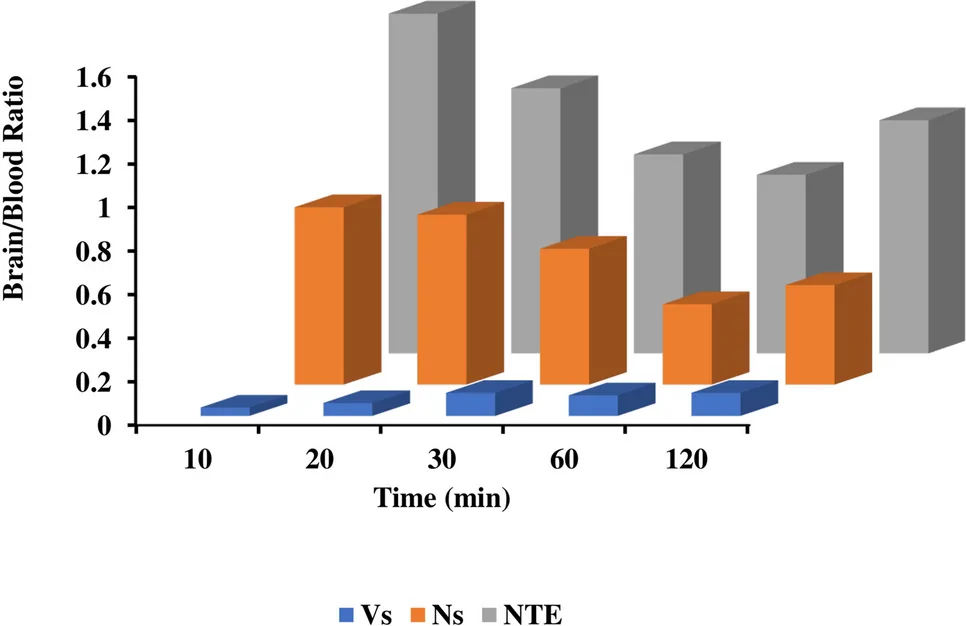
The brain/blood (B/B) ratio over time-based administration pathways

Figure 6 provides a summary of [^99m^Tc]Tc-MOD’s bio-distribution behaviour. After Vs of [^99m^Tc]Tc-MOD, there was an increase in hepatic and renal uptake (group I). Despite the systemic circulation, there was little brain uptake; this could be due to the BBB. Because the nasal delivery circumvents the blood–brain barrier and improves distribution through the olfactory and trigeminal routes, group II (Ns) demonstrated better brain targeting than group I. The presence of phosphatidylcholine and cholesterol may have contributed to vesicular rigidity and stability, which ensured persistent release, even though group III (NTE) demonstrated a booster with a sustained release effect (Fig. 6A) in comparison to groups I and III (*p* < 0.05). Additionally, phospholipids create a stable, flexible bilayer membrane that envelops medications and acts as a physical barrier against light exposure. The lipid bilayers of the nasal mucosa are fluidized by ethanol. Along with the stability of the vesicular systems that results from the presence of ethanol, this process induces membrane breakdown, allowing the soft vesicles to diffuse and discharge their contents into deeper mucosal layers [96]. Additionally, cholesterol stabilizes and improves the stiffness of the bilayer, explaining the importance of its inclusion in the formulations [97]. Furthermore, cholesterol enhanced the permeability of MOD and made it easier for the nasal mucosal membrane and the designed vesicles to interact by altering the stability and fluidity of the lipid membrane bilayer. Using Origin 9.0 SR1 data analysis and visualization software, version 90E (copyright 1991–2013, Origin Lab Corporation), Fig. 6B shows the plasma concentration of NTE, showing a decrease in systemic adverse effects compared to the solutions of both Vs and Ns (*p* < 0.05). As seen in Supplementary 1, the uptake to undesirable organs (non-target) after NTE treatment was lower than that of Ns and Vs, suggesting a decrease in unfavourable side effects as well. Furthermore, earlier studies linked a decline in systemic administration absorption to a decrease in side effects [98–100]. The averages of the different brain and blood uptakes showed significant differences between Vs, Ns, and NTE (*p* < 0.05). The NTE was significantly higher than Vs and Ns, as indicated by the B/B ratio (Fig. 7) (*p* < 0.05). Findings of bio-distribution behaviour suggest that the MOD-loaded transethosomal system may be useful for brain targeting and for either diagnosis (by labeling with [^99m^Tc]Tc) or treatment (by using just MOD-loaded transethosomes).

#### Pharmacokinetic behavior

C_max_, T_max_, and AUC_0-∞_ for blood and brain were computed to assess the pharmacokinetic behavior using the three routes of administration (Table 6). The brain C_max_ and AUC data show that MOD loaded transethosomes had greater AUC and concentration than both Vs and Ns. Continuous release was suggested by a T_max_ that was slightly delayed within blood. The pharmacokinetic profile encourages controlled release and improved absorption.

**Table 6:** Pharmacokinetic behavior of [^99m^Tc]Tc-MOD using different routes (mean ± SD, n = 3)

Formulation/route of administration/pharmacokinetic parameter	C_max_ (%ID/g)	T_max_ (min)	AUC_0–120_ (min %ID/g)	AUC_0-ꝏ_ (min %ID/g)	% Relative bioavailability
Vs [^99m^Tc]Tc-MOD/Brain	1.9 ± 0.1	30	156.9 ± 2.6	249.8 ± 79.8	
Vs [^99m^Tc]Tc-MOD/Blood	22.4 ± 1.3	10	1855.8 ± 48.9	3369.3 ± 127.4	
Ns [^99m^Tc]Tc-MOD/Brain	4.2 ± 0.2	20	344.3 ± 36.8	768.1 ± 123.6	
Ns [^99m^Tc]Tc-MOD/Blood	7.3 ± 0.55	60	693.8 ± 30.5	2653.5 ± 175.6	
NTE [^99m^Tc]Tc-MOD/Brain	5.4 ± 0.1	10	495.2 ± 18.8	3255.8 ± 490.1	424.3 ± 4.5%
NTE [^99m^Tc]Tc-MOD/Blood	5.2 ± 0.2	50	494.7 ± 12.2	2117.7 ± 139.2	107.5 ± 10.8%

*Vs* intravenous solution of [^99m^Tc]Tc-MOD, *Ns* intranasal solution of [^99m^Tc]Tc-MOD, *NTE* intranasal transethosomes administration of [^99m^Tc]Tc-MOD

DTE and DTP values were calculated for both Ns and NTE (Table 7). Partitioning time average (%DTE) of the [^99m^Tc]Tc-MOD between brain and blood [101] was 49.6% ± 3.6% and 100.1% ± 2.7% for Ns and NTE, respectively, indicating that NTE had bigger and faster transport than Ns [69]**.** On the other hand, the percentage of [^99m^Tc]Tc-MOD that was delivered straight to the brain via the trigeminal or olfactory nerve (%DTP) was 91.5% ± 0.5% and 82.9% ± 1.2% for NTE and Ns, respectively [102]. The addition of ethanol greatly fluidizes the phospholipid bilayers, making them softer and more malleable, which may account for the higher DTE and DTP values for NTE when compared to those for Ns. In addition to increasing the release rate, this structural flexibility allowed MOD to diffuse deeper from the vesicular matrix. Also edge activator play an important role in enhancing the drug delivery to brain they increase the fluidity and flexibility of the vesicle membrane, enabling the vesicles to flex and pass through tiny gaps in biological barriers. They increase the deformability of the vesicles for improved drug administration by decreasing the phospholipid bilayer’s elastic modulus [17].

**Table 7:** DTI, DTE%, and DTP% of Ns and NTE of [^99m^Tc]Tc-MOD (mean ± SD, n = 3)

Formulation/route of administration	DTE%	DTI	DTP%
Ns [^99m^Tc]Tc-MOD	49.6 ± 3.6	5.9 ± 0.4	82.9 ± 1.2
NTE [^99m^Tc]Tc-MOD	100.1 ± 2.7	11.9 ± 0.6	91.5 ± 0.5

*DTE* Drug targeting efficiency, *DTI* Drug targeting index, *DTP* Nose-to-brain direct transport percentage, *Ns* Intranasal solution of [^99m^Tc]Tc-MOD, *NTs* Intranasal transethosomes administration of [^99m^Tc]Tc-MOD

Following nasal administration, DTI shows the extent of drug targeting to the brain; higher values imply more successful brain targeting of [^99m^Tc]Tc-MOD [103, 104]. As a result, the higher DTI values for NTE than for Ns indicated that the former encouraged better drug delivery to the brain. The ability to pass through the inflexible blood–brain barrier (BBB) may be ascribed to the ultra-deformable, flexible structure, which combines the flexibility of transferosomes with the penetration-enhancing qualities of ethosomes [20]. The direct drug transport from the nose to the brain during NTE was further corroborated by the relative bioavailability percentages for the blood and brain, which were 107.5% ± 10.8% and 424.3% ± 4.5%, respectively (Table 6).

## Limitations of the study

Firstly, XRD could not be performed because the required equipment was not available at the time of the study. Moreover, the in-vivo experiments were conducted using animal models, which, while providing valuable insights, may not fully capture the complexity of human physiology and disease conditions. Furthermore, long-term stability studies were not performed.

## Conclusions

This study proved that modafinil-loaded transethosomes can be successfully engineered and optimized as an efficient and effective nanocarrier for brain targeting. The optimized formula exhibited high entrapment efficiency, nanoscale particle size, excellent colloidal stability, controlled biphasic drug release, and significantly enhanced nasal permeation. In vivo biodistribution and pharmacokinetic results confirmed superior brain accumulation, faster onset of action, and improved systemic exposure compared with the plain drug. Notably, this work is the first to quantitatively validate modafinil brain targeting by combining transethosomal nanovesicles with technetium-99 m. Collectively, these results validated our hypothesis that modafinil-loaded transethosomes represent a highly promising strategy for efficient and targeted brain delivery. This nano-platform holds substantial potential for advancing therapeutic outcomes, reducing dosing frequency, and improving patient compliance in clinical applications. The suggested system presents a logical way to get around solubility and bioavailability limitations and may facilitate future translation in intranasal CNS drug delivery.

## Supplementary Information

Below is the link to the electronic supplementary material.

ESM1 (PDF 308 KB)

## Data Availability

The datasets generated during and/or analyzed during the current study are available from the corresponding author on reasonable request.
