# Supplementary material for: Advanced modafinil-loaded transethosomes for brain targeting: development, ex-vivo permeation and radio-distribution evaluations
Source: Drug Deliv Transl Res. 2026 Apr 17;16(11):4309–29. doi: 10.1007/s13346-026-02103-y (PMC13748089; doi:10.1007/s13346-026-02103-y)
Supplement: Supplementary file 1 [file 13346_2026_2103_MOESM1_ESM.pdf]

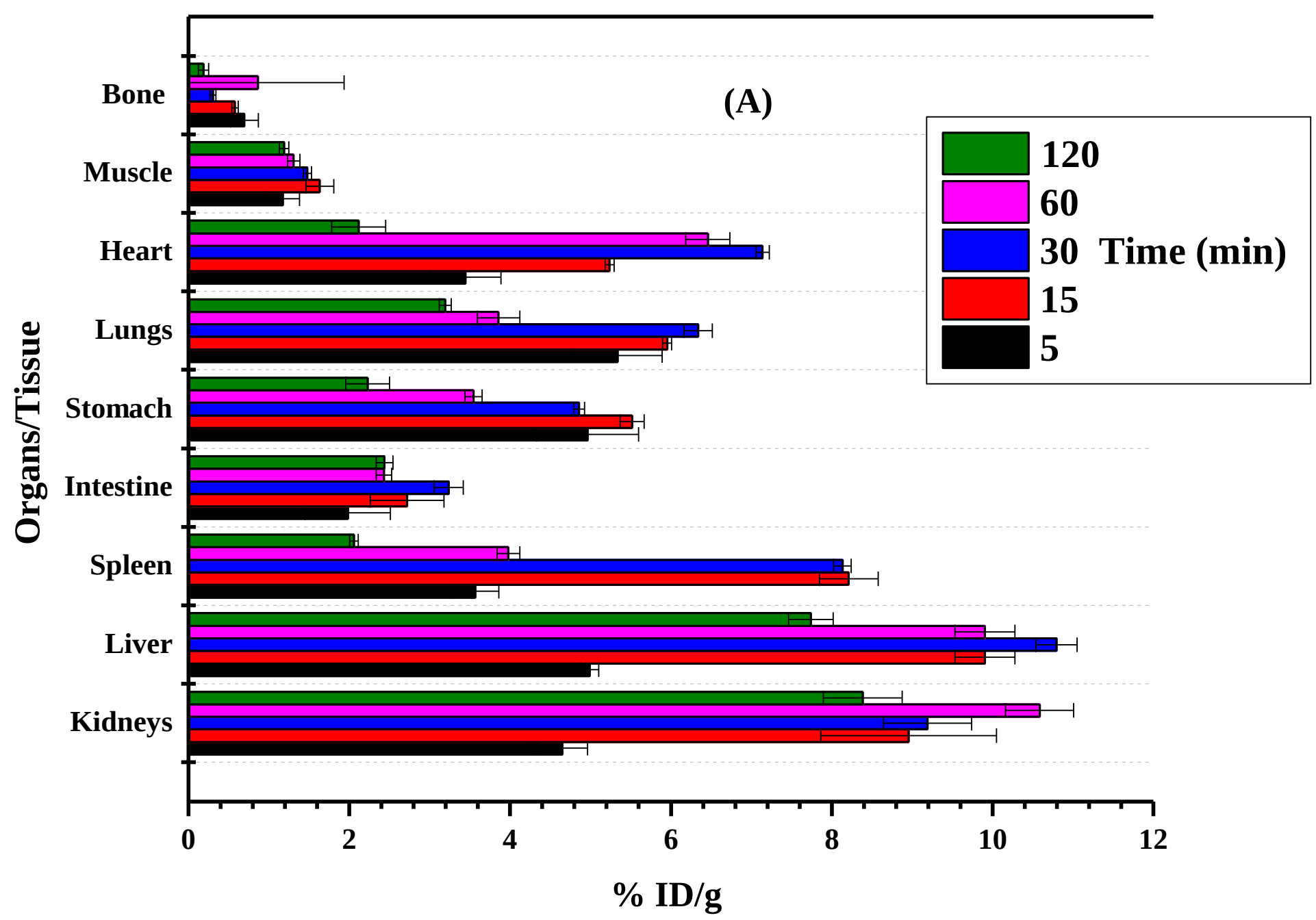

(A)
120
60
30 Time (min)
15
5
Organs/Tissue
Bone
Muscle
Heart
Lungs
Stomach
Intestine
Spleen
Liver
Kidneys
0
2
4
6
8
10
12
% ID/g

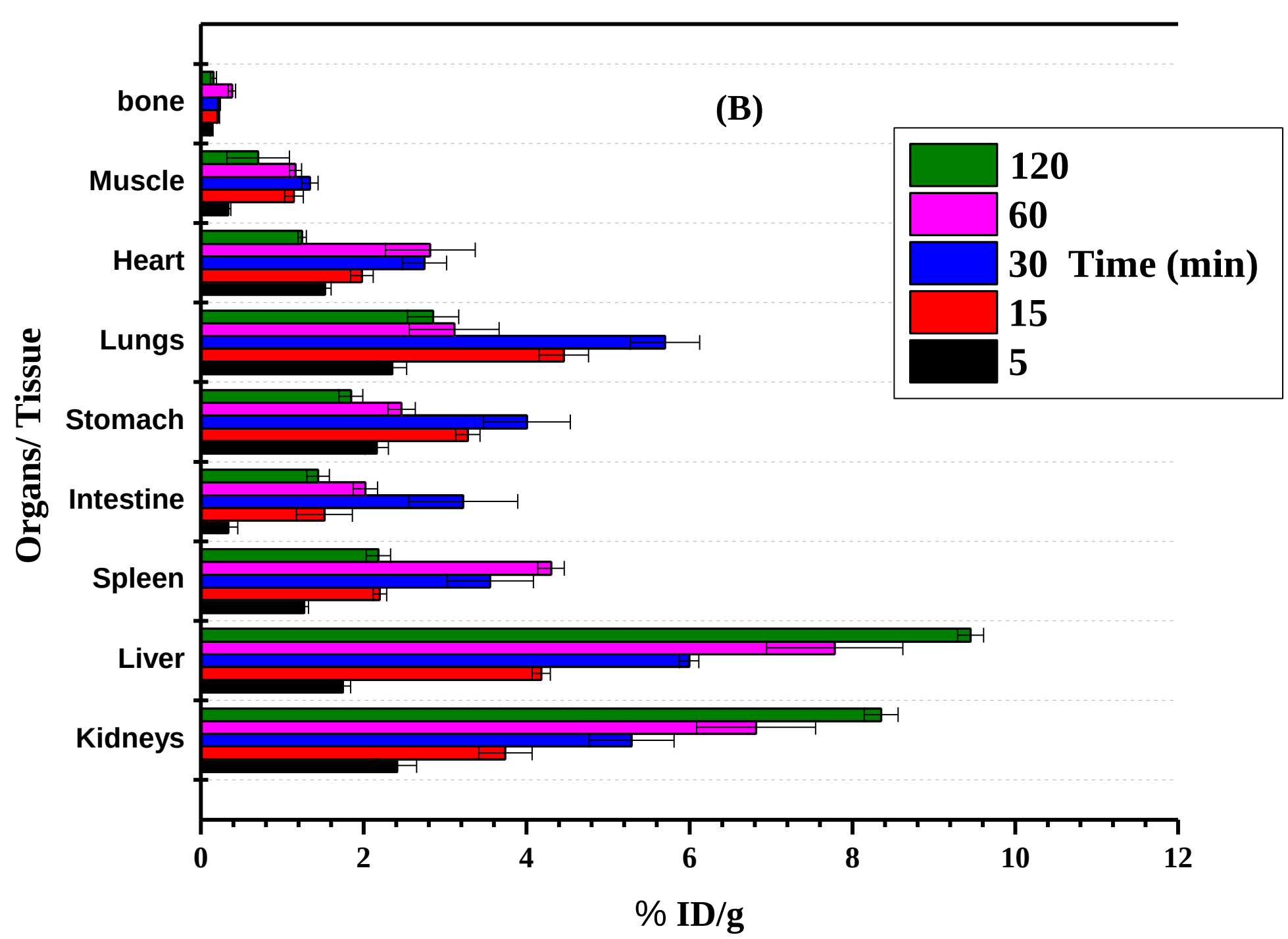

(B)
120
60
30 Time (min)
15
5
Organs/ Tissue
bone
Muscle
Heart
Lungs
Stomach
Intestine
Spleen
Liver
Kidneys
0
2
4
6
8
10
12
% ID/g

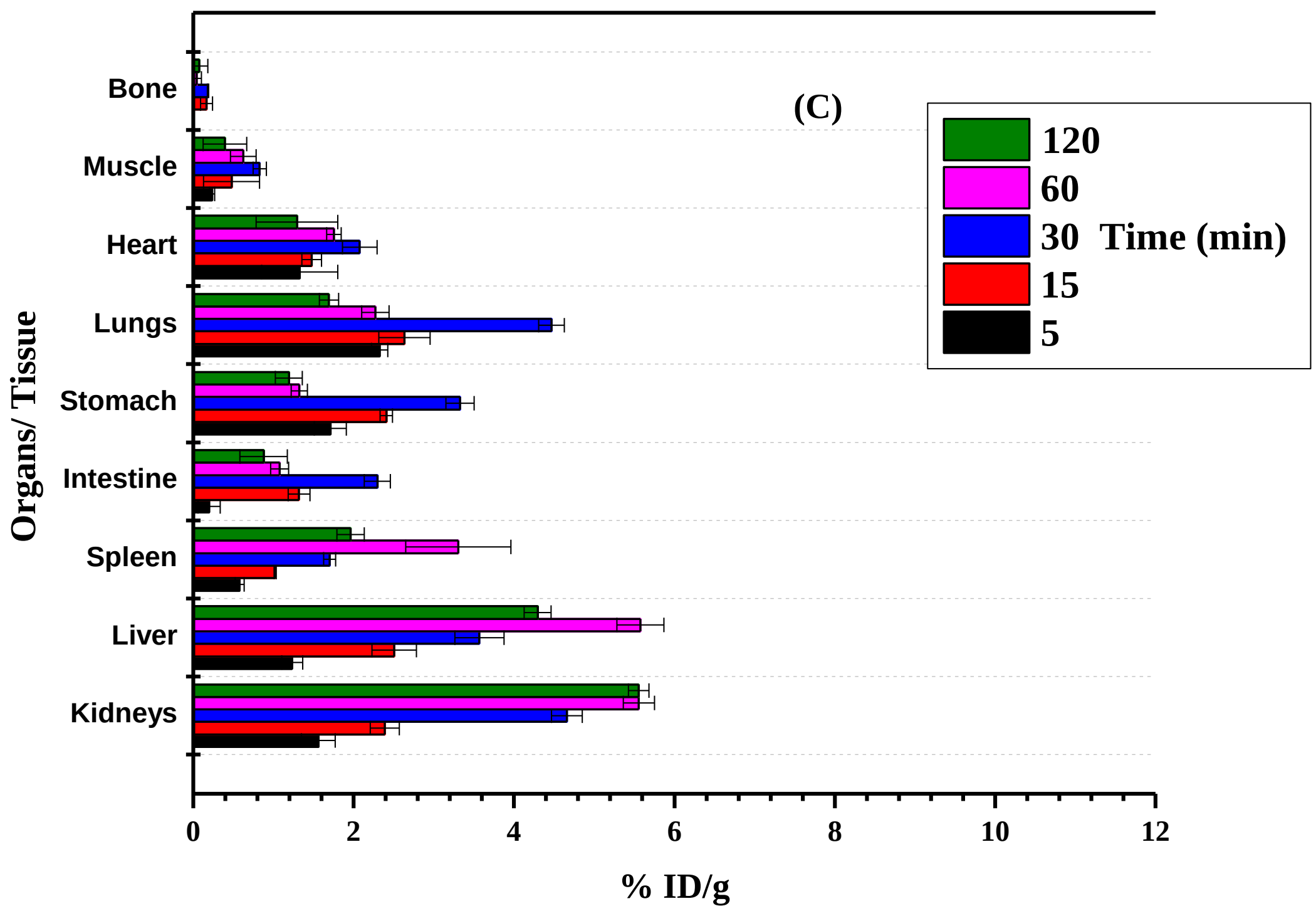

Supplementary 1: Bio-distribution behavior in different organs: (A) Vs of ^{99m}Tc-MOD, (B) Ns of ^{99m}Tc-MOD, (C) ^{99m}Tc-MOD loaded NTE
